# Mindsets impact training adaptation and high-pressure performance: beliefs and perceptions of professionals working in elite sport and mission critical teams

**DOI:** 10.3389/fspor.2026.1827074

**Published:** 2026-06-22

**Authors:** David Gray, John Kiely

**Affiliations:** Department of Physical Education and Sports Sciences, University of Limerick, Limerick, Ireland

**Keywords:** mindset, high-pressure performance, professional sport, mission critical teams, police, military, firefighting, emergency medicine

## Abstract

A critical objective in professional sports and mission critical teams such as specialist police units, military special forces and emergency medicine responders is preparing individuals, and teams, to perform optimally under intense pressure. Empirical research demonstrates that mindsets positively impact performance during stressful activities. However, no research has specifically investigated the perceived importance of mindset from individuals working in high-performance teams. In this mixed-methods study we utilised an anonymous online survey to: (1) Explore how practitioners define, perceive and understand mindset, (2) Determine the perceived importance of mindset and establish what strategies, if any, are deployed to leverage mindset in training and high-pressure performance settings, and (3) Establish the resources professional sport teams and mission critical teams allocate to the development and optimisation of mindset. This exploratory study, philosophically grounded in pragmatism, gathered the perspectives of 266 Performers and Performance Support Staff working in high-performance teams. Findings confirmed that mindsets are considered crucial to optimising performance during high-pressure situations. Mindsets are perceived to positively impact adaptation to technical, tactical and physical training. A novel finding was that a significant proportion of Performers and Performance Support Staff utilise deliberate mindset switching as a strategy to optimise performance. Participants used a multitude of definitions to define ‘mindset’ suggesting a lack of shared meaning within, and amongst, organisations. Despite mindset education being highly valued, a relatively large proportion of Performers and Performance Support Staff have never received any mindset-specific education indicating a sub-optimal and disparate approach to training provision. Findings demonstrate that mindsets are considered a pivotal component in developing, and sustaining, high-pressure, high-performance capabilities. We discuss how findings gathered from this unique cohort of professionals could improve real-world applied practice and propose future avenues for mindset research.

## Introduction

A key objective in high-performance teams such as professional sports and mission critical teams (MCTs), which includes military special operations, police tactical response units, firefighting and emergency medicine responders, is to prepare individuals and teams to perform optimally during high-pressure situations.

**Figure 1:**
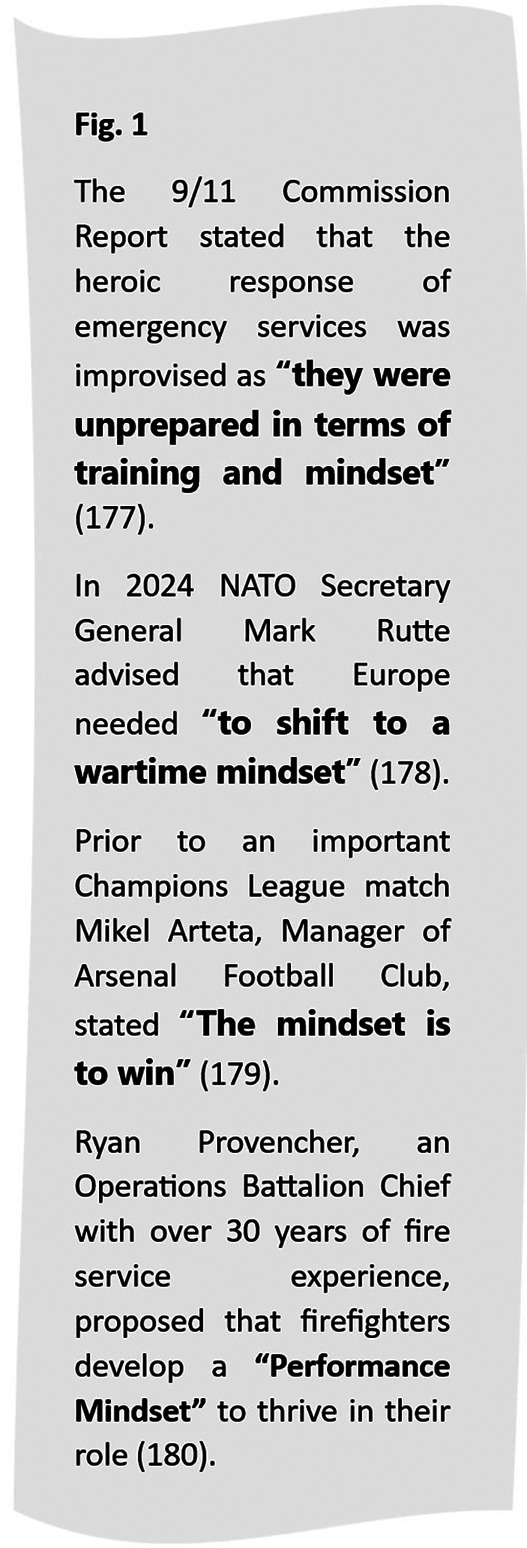
Examples of the wide-spread use of the term ’mindset’.

The term ‘mindset’ is frequently deployed in practice, and anecdotal reports suggest mindsets are a critical influence on performance within high-pressure environments (Figure 1). Empirical research demonstrates that mindset interventions trigger changes to biopsychoemotional and neurophysiological state that assist performance during high-pressure job interviews, public speaking engagements and time-constrained decision-making tasks (1–3). Military candidates holding specific mindsets in relation to stress displayed superior physical endurance and enhanced willpower during US Navy Seals special forces selection (4). Additionally mindsets positively impact an individual's resilience, grit (5), motivation and learning behaviours (6). Altering mindset facilitates adaptive changes in attentional focus, a critical cognitive construct underpinning high-pressure performance (7, 8).

Although these findings offer huge promise, only minimal mindset-related research has been conducted in high-performance teams. This is somewhat surprising as individuals and teams working in these environments are required to work at the edge of human performance. Therefore mindsets may be a critical, yet potentially underutilised, influencing factor capable of enhancing the performance capabilities of individuals, and teams, operating in high-performance settings.

Working in a high-performance team is highly demanding and exposes individuals to an array of real, or perceived, stressors (7, 9–18). Although contextual factors vary between, and amongst, professional sports and MCTs, the psychological demands are mirrored across work sectors (7, 10, 19) explaining the prevalent use of sports psychologists to deliver performance psychology support in MCTs (19). Specialist MCTs undertake specifically advanced training to prepare for volatile, uncertain, complex and ambiguous performance conditions (19–23). Individuals in specialist MCT roles are trained to make split-second decisions that may have irreversible, distressful and/or fatal consequences highlighting a magnitude of stressor and set of contextual demands that are distinct from professional sports and, in many cases, conventionally trained MCTs (10, 19, 22–25). Regardless of role, the expectation in high-performance teams is that individuals effectively manage stressors, perform optimally and deliver outcomes that are deemed successful (7, 9, 10, 12–15). Although a one-size-fits-all approach to psychological preparation is sub-optimal (23) research has identified a shared psychological architecture that underpins high-pressure performance (10, 19, 26–30) warranting the joint investigation of mindsets across professional sports and MCTs.

Exposure to high-pressure situations triggers a stress-induced up-regulation of various physiological and psychological processes that can negatively impact performance (7, 31–37). However, training, or performing, under pressurised conditions, can also promote adaptive physiological and psychological functioning (1, 38) that enhances elite performance (39–41). Therefore, it is paramount to ensure that individuals working in high-performance teams are provided the best, and most comprehensive training in which to thrive during high-pressure situations.

Although no universally agreed definition of the term ‘mindset’ has been published (42–44), there is consensus that mindsets create meaning systems (45) that are formed from an individual's implicit beliefs and core assumptions about the nature and workings of all concepts and domains in the world (4, 8, 42, 46–48). Mindsets encompass a range of domain-specific belief systems including: a growth mindset (49); a stress-is-enhancing mindset (50); a catastrophes-can-be-opportunities mindset (47); and an osteoarthritis-is-manageable mindset (51). Leading researchers propose that mindsets function as mental frames that orient attention, motivation and emotion (43, 44, 47, 50, 52–54) in a manner that mutually co-modulates physiological and neurological processes (8, 53, 55, 56). Critically, a given mindset sets a level of expectancy (57–61) that can potentially trigger adaptive, or maladaptive, changes in performance measures (53, 62). Mindsets are malleable and can be altered relatively easily, time-efficiently and inexpensively (1, 51, 63, 64). Effective mindset interventions work by changing, or refining, belief systems which, in turn, generate, shape and sustain an individual's expectations (1, 16, 44, 51, 52, 61, 65–67).

As operationalised in the present study an individual's beliefs – the constitutional elements of mindset – are understood to encompass several distinct, but interrelated, psychological processes including associations, expectancies and perceptions (45, 58, 68, 69). In summary, mindsets can be classified as general, relatively stable, abstract and evaluative sets of deeply ingrained beliefs and perceptions (61, 67). For example, growth mindset research, is grounded in the incremental theory that human abilities and attributes are malleable and can be improved (49), whereas stress mindset research focuses specifically on beliefs about the enhancing or debilitating consequences associated with stress exposure (50). Associations are formed by embedding consistently repeating patterns of experience into memories that can shape perceptions, cognitions and behaviours in real-time (70). Expectations, a psychological process critical to the neurobiological changes associated with the placebo-effect, are more transient beliefs that can be modulated by changing an individual's prediction of future outcomes or responses (44, 57). Similarly, perceptions represent a more nuanced and modifiable set of specific beliefs that can be adapted, changed or refined through life experiences and the updating of knowledge systems (67). In addition the neurobiological changes associated with the placebo phenomena are driven through the psychological processes of implicit learning and mindsets which ultimately orient individuals to a series of mindset-consistent expectations (44, 57, 61, 71). Indeed, Zion and Crum have proposed a framework in which mindsets, expectations and implicit learning are positioned as distinct but interrelated psychological processes that together activate the neurobiological mechanisms underlying the placebo effect (44).

Expectations can be considered as being the shared cognitive tissue connecting mindset constructs and the extensively studied phenomenon of placebo (i.e., anticipatory changes in the body based on an individual's beliefs on what is most likely to happen in the future) (44, 57, 61, 68, 72). This connection is not incidental as mindsets and placebos, which are evoked by mindsets (44, 71), share a common operating mechanism in that both exert effects primarily through an adjustment in expectation, which, in turn, drives neurobiological changes (69, 73). For example, placebo analgesia operates through expectancy-driven activation of the dopaminergic and endogenous opioid pathways (57), while stress mindset interventions have been shown to adjust expectations which trigger significant changes in anabolic hormone secretion (1) and cortisol reactivity (74). In both instances these outcomes reflect a ‘top-down’ modulation of neurophysiology through a belief-initiated change in expectations (50, 57, 61, 73). Therefore, an individual's beliefs and their associated expectations are deeply interconnected psychological constructs that are significant drivers of the neurobiological changes associated with mindset interventions and the placebo effect (44, 57, 61, 71, 73).

However, it is important to note that whilst the psychological constructs outlined here – stress mindset, growth mindset, expectancy effects, perceptions and placebo-related mechanisms – share a broadly belief-based architecture they represent distinct traditions of empirical enquiry (44, 61, 67, 75). Although closely related (67) these psychological constructs and processes differ meaningfully in their theoretical assumptions, measurement approaches and evidential bases. Therefore, integration carries inherent conceptual risk, and it should be recognised that boundaries between constructs are not obscured by an overarching theoretical narrative. Nevertheless, these psychological processes and constructs do converge on a shared operating principle: that belief-based re-evaluations mediate downstream cognitive, affective and neurophysiological processes.

The psychological processes and constructs outlined in this paper all play a role in forming and developing mindsets (61). Although different belief constructs should not be considered homogenous, there is a fluid interplay between processes that transitions across theoretically distinct psychological conceptual boundaries (67). It is also worth acknowledging that theoretical heterogeneity exists in the mindset research itself. Researchers have called for a new wave of mindset research that moves beyond deficit-based models of belief systems and integrates institutional factors, cultural norms and the contextual environment (76). Within stress mindset literature Jeremy Jamieson and Emily Hangen propose that more integrated cross-construct research is conducted to help build conceptual bridges between distinct, yet deeply interrelated, psychological processes (77). Despite being classified as independent psychological constructs, both mindsets and perceptions are proposed to function in a dynamic and complimentary fashion that can shape health-related outcomes (67). These insights signal that the field of mindset research has not yet converged on a unified theoretical framework which offers support to the conceptual integration employed in this investigation. Therefore, rather than proposing a unified mechanistic account, the working definition of the term ‘mindset’ adopted in this study is intended as a pragmatic, inclusive framing suited to exploratory, practitioner-focused inquiry.

We reviewed a number of definitions of mindset, published in top ranked journals, to determine the version that best suited the purposes of this investigation (42–44, 48, 50, 53, 72). The adapted definition of mindset utilised in this study is ‘*mindsets refer to our core beliefs which act as lenses that guide our future emotions, thinking, behaviours and actions’.* For the purpose of the present study the authors’ working conceptualisation of the term ‘mindset’ incorporated a deeply connected network of psychological processes including beliefs, assumptions, associations, attributions, perceptions and expectancies (44, 45, 58, 67–69, 76, 78). Each of these psychological processes can be shaped, filtered, directed and expressed through an individual's prevailing mindset which ultimately drives behaviours and neurophysiological processes that can hamper or benefit future performance (42, 51, 53, 56, 57, 61, 62, 68, 69, 79–81). This survey explored participants overarching beliefs on a range of mindset-related topics, including questions specifically focused on stress mindsets (50). This subset of questions centred on whether individuals perceive high-pressure situations as mostly helping, or mostly harming, target-task performance.

Mindsets are considered an important cognitive factor that underpins performance during high-pressure situations (9, 11, 82, 83). Holding a mindset that stress-is-enhancing (i.e., exposure to high-pressure situations can trigger adaptive responses) is beneficial to high-pressure performance (3, 50). A stress-is-enhancing mindset, or when an individual reappraises stressful situations as manageable, increases levels of dehydroepiandrosterone-sulfate (1), testosterone (38) and lowers salivary cortisol (74, 84). These neuroendocrine responses promote a more adaptive anabolic hormonal environment which improves high-pressure exam performance (38), increases resilience during challenging military training exercises (85, 86) and potentially enhances skill execution in professional athletes (87). Candidates with a stress-is-enhancing mindset displayed greater physical performance and willpower during the notoriously demanding U.S. Navy Seals selection process (4). Mindsets may also act as a psychological shield that buffers an individual from the potentially harmful effects of making skill errors during high-pressure situations (36, 88).

Updating mindsets, by shifting an individual's beliefs, assumptions and expectations, may trigger expectancy-initiated, anticipatory and prediction-driven processes that alter neural activation in the human brain (8, 31, 58, 69, 89–91). These changes in brain connectivity can activate core and peripheral physiology (69, 90, 92–97), autonomic and neuroendocrine functions (98) and alter visual attention processes (1, 8, 91). An altered biopsychoemotional state can impact cognitive performance (81), emotional state (1, 38, 43, 99), learning (63, 74), cardiorespiratory physiology (53) and subjective perceptions of pain (51, 100). Consistent with the orienting function of mindsets described above, mindsets can directly influence attentional processes – a psychological construct recognised as critical to high-pressure performance (7). Experimentally, individuals holding a stress-is-enhancing mindset demonstrated a measurable bias in visual attention, orienting preferentially towards positive rather than threatening stimuli (1) suggesting that mindset may shape the attentional lens through which high-pressure environments are perceived and interpreted. This attentional influence extends to the processing of performance errors whereby individuals holding a growth mindset exhibited significantly greater error awareness and heightened attentional engagement following mistakes, which in turn facilitated improved performance on subsequent trials (8, 91). Collectively, these findings indicate that mindsets may exert a direct and measurable influence on the allocation of attention which is particularly relevant in professional sports and MCTs where attentional control is a key determinant of performance (7). Additionally, the deliberate transition into specific mindsets, which are deemed adaptive, drives neurophysiological and neurochemical adaptation that positively influences task-specific performance (62, 69, 74).

Mindsets also impact behaviours. Older adults, suffering from knee osteoarthritis, increased physical activity levels following delivery of a specifically tailored mindset intervention (51). Preliminary evidence from a randomised control trial, in a medical setting, suggests that a mindset intervention improves patients’ adherence to challenging treatment protocols (52). Additionally, orienting individuals towards a growth mindset enhances goal-oriented effort and increases task-mastery motivational behaviours (6, 101).

Evidence-based frameworks that guide the long-term development of technical skills, psychological skills, cognitive fitness and recovery from injury in professional sport and MCT settings do exist (9, 26, 102–104). However, research on mindsets has, to date, primarily focused on the short-term impact of interventions in cohort's representative of the general population (51, 53, 60). To the authors’ knowledge, no frameworks have been published that assist in guiding an applied practitioners’ ability to develop, nurture and sustain the mindsets of individuals throughout their career in professional sports and MCTs.

From a research perspective the investigation of mindset in high-performance teams is almost completely unexplored. The overarching aim of this novel investigation was to determine the meaning, use, perceived importance and resourcing of mindset from the perspectives of Performers and Performance Support Staff (PSS) working in professional sports or MCTs. Our intention was to find relevant and practically meaningful insights that benefit the applied practices used in high-performance teams.

Firstly, despite widespread use of the term ‘mindset’ in high-performance settings, there is no agreed definition in practice. This lack of shared meaning makes it impossible to communicate clearly about how to train and use mindset effectively. Secondly, research illustrates that what people believe about stress changes what they pay attention to, how they feel, how their body responds, and how they behave under pressure. Nevertheless, we know almost nothing about how performers and PSS use mindsets day-to-day. This includes whether they change or switch mindsets deliberately or if they believe mindsets impact training adaptation and high-pressure performance. Thirdly, mindset interventions appear effective in workplaces and health settings, but it is unclear if any formal education or structured workflow processes exist within professional sports or MCTs to support mindset interventions.

To investigate these issues, this survey focused on 3 specific objectives:

1) Explore how practitioners define, perceive and understand mindset.
2) Determine the perceived importance of mindset and establish what strategies, if any, are deployed to leverage mindset in training and high-pressure performance situations.
3) Establish the resources professional sport teams and MCTs allocate to the development and optimisation of mindset.

## Materials and methods

### Experimental approach to the problem

This exploratory study utilised an anonymous online survey to investigate the perceptions and beliefs on the meaning, usefulness and impact of mindset from the perspectives of experienced practitioners working in professional sport or MCTs. A mixed-methods research design is well suited to exploratory investigations (105, 106) as gathering two streams of complimentary data can strengthen the depth and breadth of understanding of the phenomena of interest (107, 108). Qualitative and quantitative data were merged through convergent design with the integration point occurring after separate analysis of the qualitative and quantitative data (109–112). The lead researcher then determined whether quantitative findings and qualitative themes supported or contradicted each other by exploring, evaluating and considering responses as a single coherent dataset rather than a series of individual responses to specific questions (112, 113). This study was philosophically grounded in pragmatism as a key motivation of this research was to provide informative and practically impactful insights that benefit applied practitioners (114, 115). No incentives were offered to participants for completing the survey which was available for completion for 8 weeks commencing July 2024. Ethical approval was granted by University of Limerick's (Ireland) Faculty of Education and Health Sciences Research Ethics Committee (EHS REC STUDY ID: 2024_05_08_EHS).

### Participants

Non-probabilistic sampling was used to recruit a final sample of 266 participants (Performers *n* = 128, PSS *n* = 138). The sample size of 266 was deemed adequate for gathering meaningful inferences from the data (116) and exceeds, or closely aligns to, research in similar cohorts (117–123). Participant age was collected in pre-defined bands (i.e., 18–25, 26–35 years etc.), which for the purposes of this investigation was deemed an appropriate level of precision (124) and aligns to recent MCT and high-performance sport research (125–127). Participants self-identified as meeting a strict criterion-based selection (128–131) and provided voluntary consent before completing the questionnaire. A copy of the survey inclusion criteria is available in Appendix 1, Supplementary Information File. Initial recruitment was completed by direct email and text message using the research team's extensive networks (119, 132). To extend the survey reach, snowball sampling was utilised by promoting the survey on LinkedIn and X. Participants were encouraged to actively share the survey link within their relevant professional network. A copy of survey recruitment material is available in Appendix 2, Supplementary Information File.

### Survey design

This survey utilised a mixed methods design to collect qualitative and quantitative information in accordance with recognised approaches (119, 131, 133–135). Initial pilot testing was conducted with 20 non-subject matter experts to determine face validity (133–136). Content validity was established with 8, highly experienced, subject-matter experts (115). The survey was designed and hosted in Qualtrics XM and took approximately 25 min to complete. A copy of the survey, which was presented in the English language, is provided in Appendix 3, Supplementary Information File.

To assist in obtaining rich insights from participants with real-world, first-hand lived experiences, open-ended questions (survey section 3) were philosophically grounded within the ‘Critical Incident Technique’ qualitative research methodology (34, 137–140). The following working definition of mindset, upon which participants were requested to base their responses, was provided prior to completing a series of closed questions (survey section 4): “*Mindset refers to our core beliefs which act as lenses that guide our future emotions, thinking, behaviours and actions”.*

### Test-Retest reliability

To assist in establishing test-retest reliability each participant was invited to retake the survey approximately 2 weeks after initial completion (141). 23 participants (9%) submitted test-retest data (Performers *n* = 10, PSS *n* = 13). See Appendix 4, Supplementary Information File for test-retest data.

### Data analyses

Data was securely exported from Qualtrics XM to Microsoft Excel (version 2507) and cleaned. Partially incomplete submissions (i.e., missing less than 10% of data from section 3 or 4) were included in sectional analysis (142–144). Section 3 data informed the qualitative analysis. Section 4 data informed the quantitative analysis. A total of 266 data sets were analysed in section 3. 31 data sets were removed from analysis in section 4 as missing data exceeded the 10% threshold. 235 responses were analysed in section 4 (Performers *n* = 111, PSS *n* = 124).

**Figure 2:**
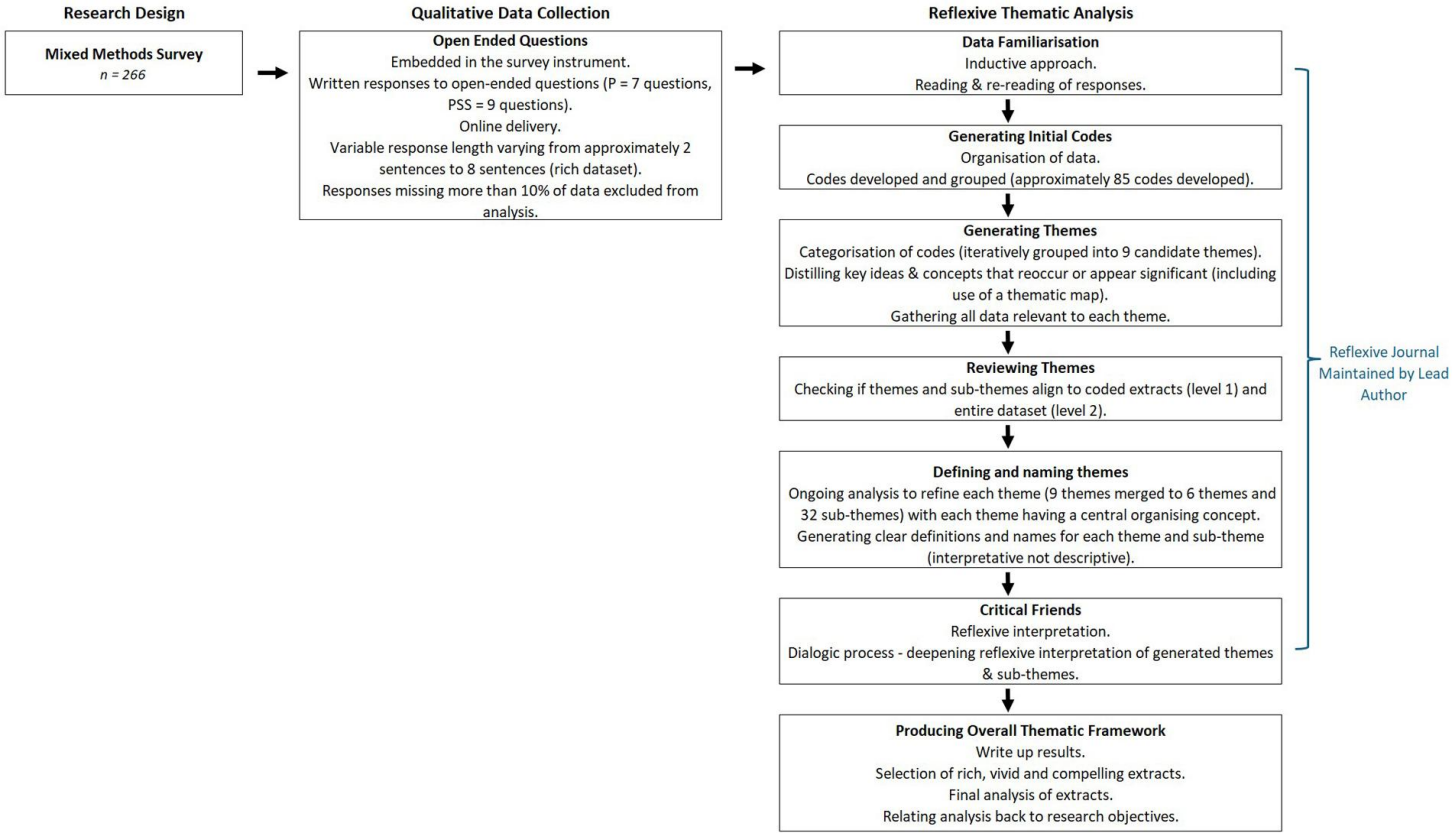
Reflexive thematic analysis approach.

Qualitative data analysis followed reflexive thematic principles which best-fit exploratory research where there is a limited understanding of the phenomena (145–147). Both researchers have extensive experience (20 + years) in physical preparation roles in professional and international sports. The lead researcher also has 5 years mindset coaching experience with professional and international athletes. In the context of reflexive thematic analysis, this professional experience is considered a valuable resource that assists in generating interpretative insights through reflexivity (113, 148) and adds methodological rigor to the findings (149). A reflexive journal was maintained throughout analysis to document observations and record the evolving interpretation of the semantic and latent meaning of the dataset (146, 150–152). The qualitative analysis approach followed a structured and systematic process which is visually depicted in Figure 2.

The key stages in the reflexive thematic analysis were data familiarisation, generating initial codes, generating themes, reviewing themes, defining and naming themes and finally producing the overarching thematic framework (146, 152, 153). To demonstrate the efficacy of each major and sub theme representative quotes and extracts are used to highlight representativeness (11). Participants are identified by role [Performer (P) or Performance Support Staff (PSS)], participant number and occupation, consistent with published practice (10).

To deepen the reasoning and transparency of the reflexive thematic analysis process, four ‘critical friends’, experienced in high-performance sport and/or performance psychology, were utilised (133, 150, 154–158). The ‘critical friends’ helped shape the lead author's thinking by constructively leveraging the beliefs, experiences and opinions of a diverse range of independent thinking partners (133). This iterative feedback process permitted the lead author and ‘critical friends’ to engage in a constructive, dialogic process that deepened the reflexive interpretation of generated themes (129, 155, 156).

In relation to quantitative analyses closed Likert-scale items (Section 4) were treated on an item-by-item basis. Descriptive statistics were selected as the primary analytical approach because the three research objectives of the investigation were exploratory and prevalence focused. Specifically, these objectives are concerned with establishing *what* practitioners believe, *how widely* those beliefs are held and *how strongly* they are endorsed, rather than testing directional hypotheses between predefined groups (105, 106). Acknowledging the ordinal structure of Likert-scale data, medians were used to summarise central tendency, inter-quartile ranges to summarise dispersion, and response proportions (%) to indicate the distribution of endorsement across scale points (159–161).

Inferential between-group testing was not a design objective of this exploratory, hypothesis-generating study. Although non-parametric comparisons such as the Mann–Whitney U or Kruskal–Wallis tests could be used in future confirmatory research to compare Performers and PSS, or sport and MCT sub-groups, such analyses were not prioritised here because the study was not powered or designed around pre-specific between-group hypotheses. Future research with targeted hypotheses, *a priori* power calculation, and appropriate adjustment for multiple comparisons would be better suited to formal inferential testing (124, 162).

Integration of quantitative and qualitative components occurred at the interpretation stages, consistent with convergent mixed-methods design (109, 110). The frequency distributions and median ratings from the closed-response questions (Section 4) were used to establish the prevalence and magnitude of mindset-related perceptions across the sample. The reflexive thematic analysis of open-ended questions (Section 3) was then used to contextualise, deepen and enrich those quantitative patterns. For example, the near-unanimous quantitative agreement that mindsets positively impact training adaptation (Table 6) was examined qualitatively to surface the specific mechanisms, conditions and caveats practitioners associate with that belief. This integrated approach is reflected in the Results and Discussion sections, where descriptive quantitative patterns are interpreted alongside participant accounts (109, 110). The data analysis and reporting adhered to published statistical reporting guidance (124) and contemporary practice in applied sport and MCT research (115, 118, 152, 163).

## Results

### General participant information

Most Performers reported their relevant work experience was in professional sport (32%), specialist police units (27%) or military special operations (10%). In relation to total years’ experience in professional sport and/or MCTs the greatest proportion of Performers had accumulated 6–10 years’ experience (30%) followed by 11–15 years (24%) and 21–25 years (14%).

The majority of PSS work in professional sport (74%) followed by specialist police units (5%) and specialist military units (4%). The greatest proportion of PSS (22%) have accumulated 16–20 years’ experience in professional sport and/or MCTs followed by 6–10 years (17%) and 21–25 years (15%).

Table 1 provides the results to the general characteristics questions contained in the participant eligibility and demographics sections of the survey.

**Table 1:** Participant characteristics.

Characteristic	Category	Performer (n =)	Performer (%)	PSS (n =)	PSS (%)
Gender	Male	118	92.2	124	89.9
	Female	9	7	14	10.1
	Non-Binary	1	0.8	0	0
Age (years)	18–25	7	5.5	1	0.7
	26–35	36	28.1	23	16.7
	36–45	46	35.9	54	39.1
	46–55	27	21.1	43	31.2
	56+	12	9.4	17	12.3
Currently active in role	Yes	95	74.2	114	82.6
*Worked in relevant role within the last 10 years	Yes	33	25.8	24	17.4
Years experience in current (or last) role	0–1	3	2.3	13	9.4
	2–4	20	15.6	24	17.4
	5–10	38	29.7	28	20.3
	11–15	34	26.6	29	21
	16–20	14	10.9	23	16.7
	20+	19	14.8	21	15.2
Total years’ experience in all relevant roles	0–5	15	11.7	16	11.6
	6–10	39	30.5	24	17.4
	11–15	31	24.2	19	13.8
	16–20	14	10.9	31	22.5
	21–25	18	14.1	21	15.2
	26–30	6	4.7	13	9.4
	31+	5	3.9	14	10.1
Work Sector(s)					
1		41	32	102	73.9
2		35	27.3	7	5.1
3		10	7.8	6	4.3
4		5	3.9	4	2.9
5		7	5.5	0	0
6		0	0	0	0
7		7	5.5	2	1.4
8		4	3.1	3	2.2
1 & 2		1	0.8	1	0.7
1,2 & 3		0	0	1	0.7
1,2 & 7		1	0.8	0	0
1 & 3		0	0	2	1.4
1,3 & 7		0	0	1	0.7
1 & 4		1	0.8	0	0
1 & 5		1	0.8	0	0
1 & 8		1	0.8	4	2.9
2 & 3		1	0.8	0	0
2,3 & 7		1	0.8	0	0
2,4 & 5		1	0.8	0	0
2 & 5		0	0	1	0.7
2,5 & 8		0	0	1	0.7
2 & 7		3	2.3	1	0.7
2 & 8		1	0.8	0	0
3 & 5		1	0.8	0	0
3 & 7		1	0.8	1	0.7
3 & 8		1	0.8	0	0
4 & 5		1	0.8	0	0
4,5 & 7		1	0.8	0	0
4 & 8		0	0	1	0.7
5 & 8		2	1.6	0	0

1, Professional Sport; 2, Specialist Police Unit; 3, Specialist Military Unit; 4, Fire Fighting; 5, Emergency Medicine; 6, Surgeon, 7, Other First Responder; 8, Other Relevant High-Pressure, High-Performance Settings, *denotes branch logic question.

### How do practitioners define, perceive and understand mindset?

#### Mindset: definition

Numerous terms are utilised by Performers and PSS to define the term mindset (Table 2). A performance psychologist working in professional football (PSS 56) supports the notion that there is a lack of shared meaning surrounding the term mindset when stating “**Mindset has the potential to be highly beneficial, but unfortunately as a term it means many different things to different people. In certain cases, it is used as a catch-all term without the required detail”**.

**Table 2:** Primary terms used to define mindset.

	Performer		Performance Support Staff	
Primary Term	Freq.	Exemplar Quote	Freq.	Exemplar Quote
“Mental approach”	14%	“Mindset is a purposefully cultivated mental approach” (P58: 2×World Cup Winning International Rugby Player)	17%	“Mental approach towards a challenge” (PSS 11: Performance Manager, International Basketball)
“State of mind”	13%	“The state of mind towards a desired goal” (P60: Undercover Police Officer)	8%	“I think it is multifactorial…state of mind” (PSS 32: Sports Scientist, Professional Baseball)
“Motivation”	12%	“Mental willingness to perform” (P56: Police Tactical Operator)	1%	“Intention/motivation to put work towards bring your best performance” (PSS 121: Professional Coach, International Rugby)
“Focused attention”	8%	“Focussed attention” (P8: Emergency Medicine Specialist)	7%	“Be here now” (PSS 22: Chief Firefighting Officer, Wildland Fire)
“Attitude”	8%	“My attitude” (P1: Team Leader Police Tactical Unit)	12%	“The attitude which you bring to any given situation” (PSS 25: High Performance Coach, International Sport)
“Beliefs & values”	8%	“A set of beliefs” (P20: Psychologist, Tier One Military Special Forces Unit)	15%	“My beliefs, values” (PSS 65: Assistant Manager, English Premier League Football)
“Expectations”	6%	“What I should look like at my best” (P37: International Rugby Player)	1%	“A particular set of expectations” (PSS 76: Cognitive Coaching Specialist: Police)
“Way of thinking”	6%	“The ability to shape the tone and character of one's thoughts” (P127: Tier One Military Special Forces Officer)	9%	“A way of thinking” (PSS 1: High Performance Consultant to Elite Professional & International Sports Teams)
“Goal-directed behaviour”	4%	“State of mind…directed at achieving a certain goal”. (P22: Police Tactical Operator)	3%	“Focus and be disciplined to execute your goal” (PSS 73: National Team Head Coach, International Rugby Sevens)
“Action”	4%	“How my inner thoughts translate to my physical actions” (P128: Tier One Military Special Forces Operator)	4%	“A mindset…guides our actions” (PSS 13: Mental Skills Trainer: Police)
“Preparation”	4%	“Being prepared both mentally and physically” (P11: Police Negotiator and Critical Incident Response Operator)	3%	“Training your mind…preparing for unknown” (PSS 19, International Netball Head Coach)
“Mental framework”	3%	“The mental frame” (P111: Team Physician, International Sport)	5%	“Cognitive frameworks” (PSS 14: Former Commander Police Counter Terrorism Unit)
“Arousal regulation”	3%	“Mindset is understanding the role you perform, the risks/dangers associated with the work, what triggers an emotional or physiological response” (P6: Police Counter Terrorist Negotiator)	3%	“The ability to choose, and maintain the appropriate levels of arousal, concentration and focus for a task” (PSS 4: National Team Head Coach, International Skiing)
“Perspective or lens”	2%	“The lens through which I am interpreting sensory inputs” (P81: Police SWAT Operator)	4%	“Perspective relative to that moment in time” (PSS 60: Strength & Conditioning Coach, International Rugby)
“Decision making”	1%	“The ability to think and make decisions on how you will act” (P28: Professional Rugby Player)	2%	“It's the capacity to receive and process information, analyse, make decisions” (PSS 17: Commander, Police Tactical Unit)

#### Mindset: perception and understanding

The majority of Performers and PSS rate their overall awareness of the concept of mindset as a 5 or above (Table 3). There was near unanimous agreement from Performers and PSS that their knowledge and understanding of mindset assists in either optimising training adaptation and high-pressure performance or their coaching, training, teaching and/or instructing respectively (Table 3).

**Table 3:** Closed-ended questions related to participants’ understanding and perceived impact of mindset.

	Performer									Performance Support Staff								
Questions	#	M (IQR)	1	2	3	4	5	6	7	#	M (IQR)	1	2	3	4	5	6	7
I consider myself to be aware of the concept of mindset	128	7 (6–7)	3.1%	0.8%	0.8%	0.8%	4.7%	22.8%	66.1%	138	7 (6–7)	3.6%	0%	0%	0.7%	5.8%	33.3%	56.5%
I have a deep understanding of mindset which optimises the coaching/training/teaching/instructing I prescribe	-	-	-	-	-	-	-	-	-	123	5 (5–6)	0%	4.1%	4.9%	8.9%	35.8%	30.9%	13.8%
My deep understanding of mindset optimises my adaptation to training and my performance during high-pressure situations	111	6 (6–7)	0.9%	1.8%	0.9%	3.6%	7.2%	42.3%	43.2%	-	-	-	-	-	-	-	-	-

#, responses; M, Median Response; IQR, Inter-Quartile Range; Values Reflect 7-point Likert Scale (1, strongly disagree; 2, disagree; 3, somewhat disagree; 4, neither agree or disagree; 5, somewhat agree; 6, agree; 7, strongly agree).

### What is the perceived importance of mindsets and what strategies, if any, are deployed to leverage mindsets in training and high-pressure performance?

#### Perceived importance of mindset: long-term development

Nearly all Performers and PSS displayed unequivocal agreement that mindset is important in relation to the long-term attainment of high levels of professional competence and elite performance status (Table 4). PSS 45 (Human Performance and Behaviour Adviser to Specialist Police Units, Former Military Special Forces Team Leader) reflected that “**From experience mindset has been the single most important factor. Mindset influences how individuals approach their profession, their beliefs in developing competency and ability to perform under pressure. It guides important behaviours associated with developing expertise and capacity”.**

**Table 4:** Closed-ended questions related to participants’ beliefs on the importance of mindset.

	Performer									Performance Support Staff								
Questions	#	M (IQR)	1	2	3	4	5	6	7	#	M (IQR)	1	2	3	4	5	6	7
How important has your mindset been in helping you reach your high level of performance?	128	7 (6–7)	0%	0%	0%	2.7%	6.3%	33.3%	57.7%	123	6 (6–7)	0%	0%	0%	3.3%	13%	38.2%	45.5%
Based on your typical workplace interactions (e.g., conversations, debriefs, operational reviews) how important is mindset to other members of your team/organisation?	108	6 (5–6.25)	0%	0%	2.8%	12%	28.7%	31.5%	25%	120	5 (5–6)	0%	0.8%	7.5%	12.5%	38.3%	23.3%	17.5%

#, responses; M, Median Response; IQR, Inter-Quartile Range, Values Reflect 7-point Likert Scale (1, not at all important; 2, very limited importance; 3, slightly important; 4, moderately important; 5, important; 6, very important; 7, critical).

#### Perceived importance of mindset: optimising training and preparation

Performers and PSS displayed almost unanimous agreement that mindsets impact how people respond, and adapt, to technical, tactical and physical training (Table 5). This perception was clearly articulated by a professional surfer (Performer 19) who stated “**Mindset is arguably more important than ability. I think mindset does 80% of the leg work and ability is 20%”**.

**Table 5:** Responses to single choice categorical questions.

**Question** *denotes branch logic question	Performer			Performance Support Staff		
	Yes	No	Unsure	Yes	No	Unsure
Does mindset impact how people respond, and adapt, to technical, tactical and/or physical training?	124 (98%)	3 (2%)	0	136 (98.6%)	0	2 (1.4%)
Is mindset important in the final few minutes BEFORE a high-pressure situation?	101 (91%)	4 (3.6%)	6 (5.4%)	110 (88.7%)	5 (4%)	9 (7.3%)
In your professional career have you ever received mindset-specific education?	71 (66.4%)	34 (31.8%)	2 (1.9%)	73 (58.9%)	45 (36.3%)	6 (4.8%)
*Would accessing mindset-specific education have helped you perform more effectively in your role?	26 (76.5%)	2 (5.9%)	6 (17.6%)	42 (93.3%)	2 (4.4%)	1 (2.2%)

The majority of Performers and PSS displayed strong agreement that mindsets trigger physiological, psychological and behavioural responses that benefit training outcomes (Table 6). As an example, PSS 76 (Psychologist: Military Special Forces Unit) stated “**Your mindsets shape what you are motivated to do and what you pay attention to. How you process the actions/words of others, how you take on feedback in training, how you think about your own performance, etc it all makes an impact on your training cycle and your performance”.**

**Table 6:** Closed-ended questions related to the perceived impact of mindset on various training factors and performance under pressure.

	Performer									Performance Support Staff								
Questions	#	M (IQR)	1	2	3	4	5	6	7	#	M (IQR)	1	2	3	4	5	6	7
*physiological adaptation to training	111	7 (6–7)	1.8%	0%	0%	1.8%	4.5%	24.3%	67.6%	124	7 (6–7)	0.8%	0%	0%	0.8%	1.6%	33.9%	62.1%
*perception of abilities & intelligence	111	7 (6–7)	1.8%	0.9%	0%	1.8%	5.4%	35.1%	55%	124	7 (6–7)	0.8%	0.8%	0%	1.6%	2.4%	23.4%	71%
*goal-oriented behaviours	111	7 (6–7)	2.7%	0%	0%	0.9%	2.7%	26.1%	67.6%	124	7 (6–7)	0.8%	0%	0%	0%	5.6%	32.3%	60.5%
*motivation	111	7 (6–7)	1.8%	0%	0.9%	0%	6.3%	20.7%	70.3%	124	7 (7–7)	0.8%	0.8%	0%	0.8%	3.2%	16.9%	77.4%
*visual scanning & perception	111	6 (6–7)	0.9%	0%	0%	4.5%	12.6%	34.2%	45%	124	6 (6–7)	0%	0.8%	0.8%	4%	12.9%	33.1%	44.4%
*decision-making	111	7 (6–7)	1.8%	0%	0%	0.9%	1.8%	29.7%	65.8%	124	7 (6–7)	0.8%	0%	0%	1.6%	4.8%	25.8%	66.9%
*focus & attention	111	7 (6–7)	2.7%	0%	0%	0.9%	1.8%	31.5%	63.1%	124	7 (6–7)	0.8%	0%	0%	1.6%	6.5%	16.9%	71.8%
*technical skill execution	111	7 (6–7)	0.9%	0%	0.9%	0.9%	8.1%	26.1%	63.1%	124	7 (6–7)	0.8%	0%	0%	4.8%	9.7%	25.8%	57.3%
*physiological & psychological response to high-pressure situations	111	7 (7–7)	1.8%	0%	0%	0%	1.8%	19.8%	76.6%	124	7 (7–7)	0.8%	0%	0%	0.8%	3.2%	15.3%	78.2%
Mindsets trigger physiological and behavioural responses that can either be adaptive or maladaptive to training and performance outcomes.	111	7 (6.5–7)	3.6%	0%	0%	0%	0.9%	20.7%	74.8%	124	7 (6–7)	4.8%	0.8%	0%	0.8%	5.6%	20.2%	67.7%

#, number of responses; M, Median Response; IQR, Inter-Quartile Range; Values Reflect 7-point Likert Scale (1, strongly disagree; 2, disagree; 3, somewhat disagree; 4, neither agree or disagree; 5, somewhat agree; 6, agree; 7, strongly agree). * items completed as “Mindsets can positively impact [x]”.

Respondents displayed high levels of agreement that mindsets positively impact a multitude of training and performance factors (Table 6). Most Performers and PSS rated the importance of their mindset as a 6, or above, in relation to positively impacting physiological adaptation to training, motivation, goal-directed behaviours, focus and attention, decision making, visual scanning behaviours, technical skill execution and perception of abilities and intelligence (Table 6).

#### Perceived importance of mindset: leading into high-pressure situations

91% of Performers and 89% of PSS considered mindsets to be important in the minutes leading into a high-pressure situation (Table 5). Performer 87 (FBI Special Agent) emphasised that it's “**those moments right before you launch that my senses were most acute. If I wasn't in the right mindset, I risked making a mistake which could have catastrophic results”.**

#### Perceived importance of mindset: during high-pressure situations

A large proportion of Performers and PSS considered mindsets to have an important role in optimising technical, tactical and physical performance during high-pressure situations (Table 7). An Olympic athlete (Performer 114) stated “**mindset has been the most impactful piece. Physical is simple, do it, and you’ll get out what you put in. It's the mind that either makes or breaks people in pressure situations”**.

**Table 7:** Closed-ended questions related to the perceived impact of mindset during high-pressure situations.

	Performer									Performance Support Staff								
Questions	#	M (IQR)	1	2	3	4	5	6	7	#	M (IQR)	1	2	3	4	5	6	7
*technical skill execution	111	6 (5.5–7)	0%	0%	4.5%	0.9%	19.8%	36%	37.8%	124	6 (5–7)	0.8%	1.6%	0%	7.3%	20.2%	36.3%	33.1%
*tactical performance (e.g., decision-making, problem solving)	111	6 (6–7)	0%	0%	0.9%	1.8%	9.9%	37.8%	49.5%	124	6 (6–7)	0%	0%	0%	1.6%	15.3%	38.7%	43.5%
*physical performance	111	6 (5–7)	0%	0.9%	0.9%	5.4%	25.2%	29.7%	37.8%	124	6 (5–7)	0%	0.8%	2.4%	8.1%	20.2%	34.7%	33.1%

#, responses; M, Median Response; IQR=Inter-Quartile Range; Values Reflect 7-point Likert Scale (1, not at all important; 2, very limited importance; 3, slightly important; 4, moderately important; 5, important; 6, very important; 7, critical). *items completed as “During high-pressure situations is mindset important in optimising [x]”.

In specific relation to optimising technical skill execution during high-pressure situations most Performers and PSS rated the importance of mindset at a 5 or above (Table 7). This perception was reinforced by Performer 63 (Emergency Aeromedical Specialist) who states “**my technical execution is where I believe mindset has the most potential impact…my ability to perform ‘high acuity, low occurrence’ procedures requires me to believe in myself in challenging settings”**.

Performers and PSS displayed almost unanimous agreement on the importance of mindset in relation to successfully completing challenging cognitive tasks such as problem solving and decision making during high-pressure situation (Table 7). Performer 17 (Firefighter) emphasised that “**Your mindset allows you to trust in yourself and trust in the decisions you make”**. Performer 89 (Polic SWAT team Leader) agreed that “**Decision making is the most important thing in pressure situations. My mindset allows for critical thinking in these situations”**.

The majority of Performers and PSS rated the importance of mindsets at a 5 or above in relation to optimising physical performance during high-pressure situations (Table 7). PSS 74 (Strength & Conditioning Coach: International & Olympic Hockey) stated “**Mindset is what allows all the physical performance to unfold in high pressure environments”**.

#### Mindset optimisation strategies: deliberate mindset switching

85% of Performers (Table 8, median response 6, IQR 5-7) and 83% of PSS (Table 8, median response 6, IQR 5.75-7) reported using deliberate mindset switching, as a strategy to optimise performance relative to the task demands. PSS 91 (Army Physical Training Instructor) emphasised that “**Mindset is the capacity to adopt or implement a specific mental/psychosocial framework to facilitate performance. It is a conscious shift in psychology/physiology, aiming to mitigate environmental/external influences and aid performance”**. Similarly, Performer 126 (Emergency Medicine Specialist) stated that “**Mindset is always present and there are different types of mindsets that can be deliberately chosen”**.

**Table 8:** Closed-ended questions related to participants’ use of deliberate mindset switching.

	Performer									Performance Support Staff								
Question	#	M (IQR)	1	2	3	4	5	6	7	#	M (IQR)	1	2	3	4	5	6	7
Based on the specific demands of a task, or situation, I will deliberately transition/switch between different mindsets to optimise my performance	111	6 (5–7)	3.6%	2.7%	5.4%	3.6%	18%	37.8%	28.8%	124	6 (5.75–7)	1.6%	4%	2.4%	4%	12.9%	37.1%	33.1%

#, responses; M, Median Response; IQR, Inter-Quartile Range; Values Reflect 7-point Likert Scale (1, strongly disagree; 2, disagree; 3, somewhat disagree; 4, neither agree or disagree; 5, somewhat agree; 6, agree; 7, strongly agree).

### Establish the resources professional sports and MCTs allocate to the development and optimisation of mindset

#### Provision of mindset-specific education

66% of Performers and 59% of PSS have completed mindset-specific education during their professional career (Table 5). 32% of Performers and 36% of PSS have never received, nor undertaken, any form of mindset-specific education during their professional career (Table 5).

#### Perceived Impact of Mindset-Specific Education

The majority of Performers rated the impact of mindset-specific education as a 5 or higher in relation to improving technical, tactical and physical training outcomes as well as their ability to perform during high-pressure situations (Table 9). Most PSS (83%) rated the impact of mindset-specific education on their coaching, teaching, training or instructing as a 5 or above (Table 9).

**Table 9:** Closed-ended questions related to impact of mindset-specific education.

	Performer									Performance Support Staff								
Questions	#	M (IQR)	1	2	3	4	5	6	7	#	M (IQR)	1	2	3	4	5	6	7
* optimising your adaptation, and response to technical skills training	72	6 (5–7)	0%	1.4%	1.4%	13.9%	29.2%	26.4%	26.4%	-	-	-	-	-	-	-	-	-
* optimising your adaptation, and response to tactical training (e.g., decision-making, problem solving)?	72	6 (5–7)	0%	1.4%	1.4%	8.3%	20.8%	30.6%	36.1%	-	-	-	-	-	-	-	-	-
* optimising your adaptation, and response, to physical training?	72	5 (4–6)	0%	6.9%	6.9%	12.5%	27.8%	22.2%	22.2%	-	-	-	-	-	-	-	-	-
*optimising your performance during high-pressure situations?	71	6 (5–7)	0%	1.4%	2.8%	8.5%	18.3%	32.4%	36.6%	-	-	-	-	-	-	-	-	-
* improving your coaching/teaching/training/instructing?	-	-	-	-	-	-	-	-	-	72	6 (5–6)	0%	1.4%	4.2%	11.1%	27.8%	31.9%	23.6%

#, responses; M, Median Response; IQR, Inter-Quartile Range; Values Reflect 7-point Likert Scale (1, not at all important; 2, very limited importance; 3, slightly important; 4, moderately important; 5, important; 6, very important; 7, critical). *items completed as “How important was this mindset-specific education in [x]”.

#### Format and frequency of mindset specific education

Responses to the question ‘*Could you briefly describe the format, and frequency, of the mindset-specific education you received’* revealed that knowledge on mindset is primarily accrued through 10 educational pathways (Table 10).

**Table 10:** Educational pathways to develop mindset-specific knowledge.

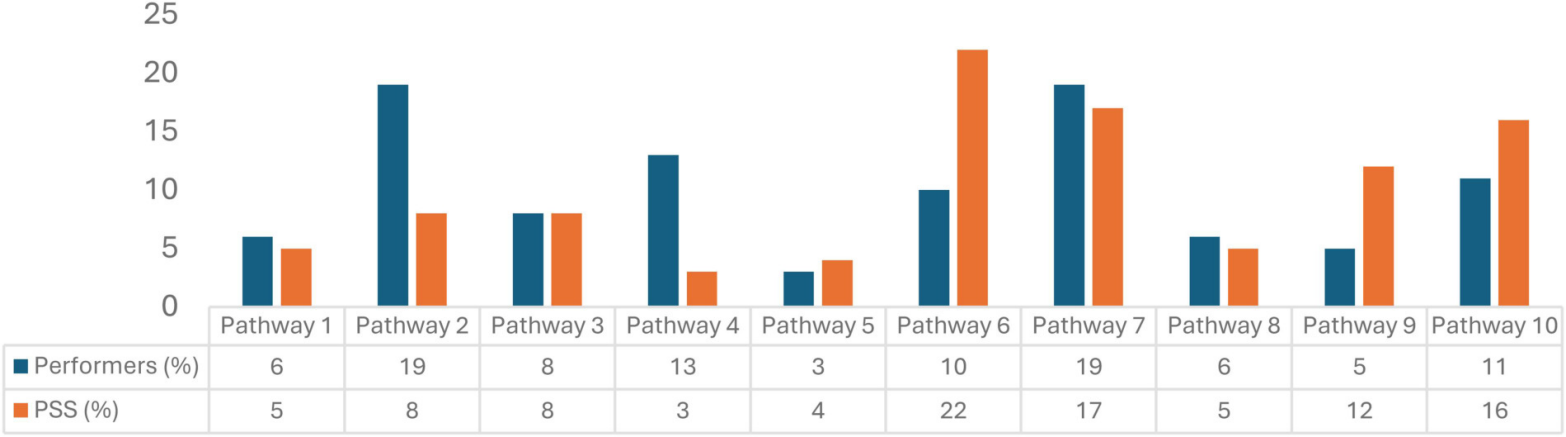

Education Pathway 1, Full-time embedded resource in organisation; 2, Part-time (2–3 days/week) embedded resource in organisation; 3, Part-time (1 day/week) embedded resource in organisation; 4, Organisation provides access to an external expert (1 day/month); 5, Self-organised access to an expert with no affiliation to organisation; 6, Completed during initial training, specialised training courses and/or continued professional development; 7, Attend workshops containing mindset information (1 × 6–12 months); 8, Informal education (e.g., mentor); 9, Completed mindset-related education during academic studies prior to commencing professional role, 10, Self-development (e.g., podcasts, books).

## Discussion

This novel study aimed to survey the opinions and beliefs of Performers and PSS, working in professional sport or MCTs, on the meaning, usefulness, perceived impact and resourcing of mindset within their performance environment. The discussion is subdivided into 3 sections to systematically address the key objectives of this investigation.

### How do practitioners define, perceive and understand mindset?

The term mindset is deeply embedded in the daily vernacular of professional sport organisations and MCTs (Tables 2, 4). However, participants used a wide range of terms to define mindset (Table 2). The most common descriptors included ‘mental approach’ (14% Performers, 17% PSS), ’state of mind’ (13% Performers, 8% PSS), ‘beliefs and values’ (8% Performers, 15% PSS), and attitude’ (8% Performers, 12% PSS). This finding illustrates that practitioners think about mindset in multiple ways, while also clearly revealing practitioners’ beliefs that there is a lack of shared vocabulary within, and amongst, organisations.

The multitude of terms used to define mindset (Table 2), which were collected prior to the authors sharing the working definition of the term mindset used in this survey, indicates there may be a lack of clarity on the meaning of mindset within professional sports and MCTs. Despite this ambiguity, 85% of Performers and 79% of PSS consider mindset to be important at an organisational level as it is frequently mentioned during interactions such as conversations, debriefs and mission planning sessions (Table 4). This finding is deemed highly important, as creating a shared meaning system of key terminology improves communication and performance feedback (164) whilst also benefitting individual and team performance through improved team collaboration (165). However, when shared meaning systems are lacking in high-performance teams, it can lead to individuals forming their own tacit interpretations and assumptions that can increase misunderstandings, promote conflict and negatively impact team dynamics in relation to goal achievement (166).

Based on the findings of this investigation it appears likely that many discussions that reference mindset are most likely generating different meanings, intentions and expectations amongst team members and work colleagues. This key finding highlights a potentially significant blind spot in applied practice. As a matter of urgency high-performance teams should prioritise the optimisation of formal and informal communication frameworks, by ensuring they have disseminated an accurate, clearly defined and understood working definition of mindset amongst all relevant personnel. This suggested intervention has the potential to augment training and performance outcomes in high-performance teams.

### What is the perceived importance of mindset and what strategies are deployed to leverage mindset in training and high-pressure performance settings

Mindsets are perceived by practitioners to be a critical psychological construct underpinning high-pressure performance capability and the development of role-relevant technical, tactical and physical skillsets. Performers and PSS displayed near-unanimous agreement that mindsets are crucial in relation to both training adaptation and high-pressure performance (Tables 3, 5–7). Most respondents reported strong levels of agreement regarding the perceived positive impact of mindsets in relation to physiological adaptation to training, technical skill execution, decision-making, motivation, attention and physiological and psychological responses during high-pressure situations.

A novel, and practically interesting finding, in relation to the use of applied mindset strategies, was the significant proportion of Performers and PSS who report utilising deliberate mindset switching to optimise their performance relative to task demands (Table 8). Three important caveats should be noted in relation to this finding before its implications are considered. Firstly, the neurophysiological and psychological mechanisms proposed to underlie deliberate mindset switching remain entirely speculative within the present context, as this study was not designed to assess underlying mechanism. Secondly, the behavioural effectiveness of this strategy has not been empirically established with the present data reflecting practitioner beliefs and perceived utility rather than directly measured performance outcomes. Consequently, applied recommendations regarding deliberate mindset switching should be treated with caution until prospective experimental studies provide direct evidence of efficacy.

With these caveats clearly acknowledged the reported prevalence of deliberate mindset switching in nonetheless a meaningful finding from an exploratory perspective. Drawing cautiously on prior laboratory-based research – whilst recognising findings were not generated in the present study – the applied strategy of deliberate mindset switching may, theoretically, be utilised to consciously influence, shape or redirect the brains’ generative model to trigger a set of anticipatory and prediction-driven processes that are adaptive to performance (31, 62, 167). Although the benefits of this applied mindset strategy remain to be empirically established prior research does suggest that mindsets augment high-pressure performance by triggering a series of biopsychoemotional and neurophysiological changes that occur in a direction, and magnitude, that reflects the “updated” mindset and associated expectancies (1, 48, 50, 52, 53, 60, 68, 80, 81). Additionally, existing research suggests that the brain's predictions, which are partially formed through mindsets, shape an individual's perception and attention in relation to the processing of incoming visual information (8, 78, 168). Theoretically, this process may explain why individuals holding specific mindsets are more effective at maintaining attentional focus on task-relevant stimuli following errors in performance (8, 91). As attention is a critical cognitive factor underpinning high-pressure performance (7), the intriguing, and previously undocumented, practitioner reports of deliberate mindset switching provides a potentially significant insight into the applied mindset strategies used by elite practitioners. However, these mechanistic accounts extend well beyond the evidential scope of the present survey and should be regarded as hypothesis-generating rather than explanatory.

Pending empirical validation, deliberate mindset switching could potentially benefit Performers and PSS who are required to rapidly transition between distinct performance-related tasks. As an example, a tactical police officer may prioritise one mindset to trigger predictions that elicit a biopsychoemotional and attentional state that is deemed the most adaptive to apprehend a violent offender during a direct-action assault. The same officer may then switch mindset as they transition to their next task, which may have significantly different contextual demands, such as providing medical attention to injured by-standers. To advance applied practice empirical research investigating the mechanistic underpinnings, performance impact and the most effective, and efficient, mindset switching strategies is strongly recommended before this finding is applied in practice.

This study also aimed to determine whether mindsets are deemed important in relation to training adaptation and outcomes. Practitioners reported near-unanimous agreement that mindsets are considered an important factor underpinning the completion of long-term training that progressively develops an individual's level of professional competency (Table 4). Additionally, most Performers and PSS report believing that mindsets are associated with physiological and behavioural responses that benefit a host of training outcomes that are advantageous to elite performance (Table 6). It is important to note that these are practitioner perceptions rather than directly measured outcomes, and caution is warranted in interpreting them as evidence of a causal relationship between mindset and training adaptation. With this interpretative boundary clearly stated, these perceptions are nonetheless broadly consistent with existing research findings. Attaining a professional role in sports or MCTs typically requires physical effort, ongoing learning, resilience, grit, willpower and motivation across multi-year training phases (4, 9, 10, 152, 169). Although it was beyond the scope of this investigation to assess specific mindsets, prior research suggests that displaying the afore mentioned psychological traits and self-regulation capacities are associated with growth mindsets (5, 6, 49, 101, 170), stress-is-enhancing mindsets (1, 4) and harnessing the self-fulfilling nature of mindsets (68, 81, 171).

The potential use of other, yet to be determined, mindsets that may specifically augment adaptation to technical, tactical and physical training requires further investigation. It may be beneficial for research teams to determine the prevalence of various mindsets, at different time points, to assess if, and how, these mindsets may evolve during a long-term career. Longitudinal research may ultimately allow high-performance teams to implement specific mindset interventions that help each Performer and PSS be best-prepared, from a mindset perspective, across their career trajectory. These findings offer applied practitioner's meaningful opportunities to develop and nurture the mindsets of aspiring, newly recruited and experienced Performers and PSS in high-performance teams.

### Establish the resources professional sport teams and MCTs allocate to the development and optimisation of mindset

This investigation highlights the significant gaps that exist in the current approach to training and educating of mindset in professional sports and MCTs. Despite mindset education being highly valued (Table 9), 32% of Performers and 36% of PSS have never received any mindset-specific education (Table 5). Among those who have, there is an enormous disparity in the focus, format and quality of these educational interventions (Table 10). It must be noted that these findings reflect practitioner perceptions and self-reported experiences rather than an objective audit of educational provision and therefore should be interpreted accordingly.

Although Performer and PSS roles can vary in terms of perceived status within an organisation, all Performers and PSS, to a greater or lesser degree, have influence on other people. Ensuring all team members are appropriately educated on mindset is critical as individuals, holding influential roles mediate and shape the overall mindset culture of an organisation (45, 172–174) which impacts the mindsets of others in a beneficial or detrimental manner (175). As a 3-hour mindset education program was deemed beneficial in a specific MCT setting (66) there is preliminary support underpinning the recommendation that high-performance teams ensure all relevant staff are, at a minimum, provided a base level of mindset-specific education. For example, ensuring Performers and PSS understand the self-fulfilling nature of mindsets (68), the power of expectations (44) and the impact of presenting information in a format that positively influences mindset (51) could theoretically benefit individual, team and organisational performance.

The disparity of educational formats used across professional sports and MCTs suggests, from the perspectives of the subjects involved in this study, there is an urgent need for organisations to evaluate their current approach to optimising mindset. Findings from this investigation indicate there is an apparent lack of any coherently structured education model (Table 10), which, in combination with the reported uncertainty around the meaning of the term mindset (Table 2), suggests that the approach to educating, training and coaching mindset is potentially, in many professional sports organisations and MCTs, sub-optimal at best. Where necessary organisations should, if possible, invest in developing role-relevant and evidence-based mindset resources for Performers and PSS. Additionally, the development of validated frameworks to guide applied practice is deemed beneficial in many high-performance settings (9, 102, 104). It is suggested that future research specifically investigates the mindset-related education and training provision utilised in professional sports and MCTs. However, based on the findings of this investigation it is suggested that researchers, Performers and PSS collaborate and co-create a validated mindset development and training framework to guide applied practice. As mindsets are perceived to have a positive impact on a host of training and performance factors (Tables 3, 5–7) it is plausible that any high-performance team, willing to innovate and embed mindset-informed education and training principles could generate a performance advantage.

## Conclusion

This investigation had three specific objectives. Firstly, we aimed to understand how practitioners define, perceive and understand mindset. Secondly, we wished to determine whether mindsets are deemed important and, if so, what strategies experienced professionals deploy to leverage mindset. Thirdly, we wanted to establish the level of resources high-performance teams allocate to the development and optimisation of mindsets.

This paper is the first to describe the beliefs and perceptions of Performers and PSS regarding the meaning, use, perceived importance and resourcing of mindset in professional sport and MCTs. It is clear from this research that practitioners perceive mindsets as positively impacting performance during high-pressure situations as well as adaptation to technical, tactical and physical training. It is particularly noteworthy that Performers and PSS report using deliberate mindset switching as an applied strategy to optimise performance relative to task demands. However, this finding is exploratory and requires direct empirical investigation to establish its effectiveness and underlying mechanisms. A multitude of terms were used by respondents when defining the term mindset suggesting that organisations could benefit from clearly establishing the meaning of mindset amongst Performers and PSS. Despite mindset-specific education being highly valued there is huge disparity in the methods high-performance teams are reported to use to educate their team members. In addition, a relatively large proportion of Performers and PSS report receiving no formal mindset education. The development of a co-created, structured, coherent and evidence-based mindset educational and training resource that guides applied practices would appear to be beneficial.

Key take home messages for practitioners:

- Practitioners perceive mindsets as positively impacting performance under pressure and training outcomes.
- Deliberate mindset switching is considered by practitioners to be a useful strategy to optimise high-pressure performance.
- Clarifying the meaning of the term ‘mindset’ to team members will generate a shared meaning system that could increase collaboration, improve communication and enhance team dynamics.
- Developing and embedding a validated mindset training framework into a high-performance team's day-to-day environment could benefit individual, team and organisational performance.

The recommendations made within this paper should stimulate discussion and critical thinking amongst, and between, professional sports organisations and MCTs. Although some teams, particularly in professional sport, may be direct competitors there is an opportunity for organisations, and applied researchers, to pool resources, grow cohesion and spread knowledge that further explores the perceived impact of mindsets on training and high-pressure performance.

### Future research directions and practical applications

This study offers a number of contributions that could augment applied practices and guide future mindset-related research. The finding that deliberate mindset switching is widely reported within high-performance teams is of applied relevance, although its effectiveness and mechanistic underpinnings remain to be empirically established. Researchers should initially investigate the effectiveness and impact of this applied mindset strategy. If mindset switching augments performance, disseminating information on the most effective strategies to transition between, or to consciously induce specific mindsets, may benefit individuals working in high-performance teams.

Leaders of high-performance teams should ensure that the term mindset is clearly defined, and understood, amongst team members. Where necessary, a working definition of mindset should be provided so all Performers and PSS can communicate effectively through a shared meaning system.

Findings revealed the perceived importance of mindsets in relation to training outcomes and performance during high-pressure situations. Organisations may wish to consider assessing the growth and stress mindset characteristics of current, or future, team members. Both growth mindsets (176) and stress mindsets (4) can be measured through validated scales. Assessing Performers and PSS mindsets and, where necessary, implementing mindset interventions may be an effective strategy to further enhance training outcomes and high-pressure performance. It is also apparent, from the authors’ perspective, that future research could focus on the development of a validated mindset training framework. A co-created framework may assist in guiding the structured, and progressive, delivery of mindset interventions, applied performance strategies and mindset-related educational content within high-performance teams. Embedding a mindset training framework within high-performance teams may benefit aspiring, newly recruited, mid-career and experienced Performers and PSS. It is suggested that high-performance teams embed, or at a minimum engage with, a mindset expert to lead, manage and support the integration of a mindset framework as part of a multi-disciplinarian human high-performance program.

Finally, results revealed that completing mindset-specific education is perceived to benefit a host of training outcomes as well as performance during high-pressure situations. Therefore, based on the findings of this investigation, it appears paramount that professional sports organisations and MCTs ensure that appropriate investment is allocated to the development, and sustainment, of team members mindset knowledge. A practically significant area of future research could involve academic experts engaging with Performers and PSS to collaboratively develop a syllabus of useful, meaningful and role-relevant educational content.

### Strengths

This research adds a number of novel and practically significant findings that could augment the mindset-related practices currently utilised in high-performance teams.

A major strength of this investigation was the diverse range of elite-level participants recruited. The novel and comprehensive insights gathered from 266 high-performers, experienced in a multitude of high-pressure settings, enhances the relevance of findings across high-performance teams and should be considered a unique strength of this investigation.

The study design adhered to best-practice guidelines and demonstrated a high-level of methodological rigor that goes beyond similar research conducted in elite performance settings. Piloting the survey, utilising experts to establish content validity and reporting on test-retest reliability follows best-practice guidelines. Additionally, engaging in an iterative and constructive dialogic process with 4 critical friends deepened the author's reflexive interpretation of qualitative findings. Furthermore, openly and transparently acknowledging the authors’ extensive experience in professional and international sport is deemed advantageous and further strengthens the reflexive thematic analysis approach. The mixed-method design and use of open-ended questions, philosophically grounded in the critical incident technique, allowed a wealth of information to be gathered. Providing a working definition of the term mindset, which was used to objectify participants responses, in Section 4 of the survey, strengthens findings due to the multiple, and variable, definitions of mindset respondents presented.

### Limitations

The results should also be considered in respect of a few limitations. As the present study relied on self-report data the findings of this investigation reflect practitioner perceptions and beliefs rather than empirically tested, direct measures of physiological, behavioural or neurocognitive outcomes associated with mindsets.

The limited test-retest sample size of 9% may have skewed the generalisability of the reliability estimates as re-testers may have differed systematically from non-re-testers.

The impact of response bias in this investigation is worth considering as non-probabilistic sampling was used to recruit subjects. Participants in this research may have already held strong opinions regarding the importance of mindset which motivated their involvement. Therefore, the findings of this investigation may not necessarily generalise to all Performers and PSS in professional sports and MCTs.

Although the qualitative data analysis process adhered to best-practice guidelines the practical constraints faced by the research team need acknowledged. Utilising a second independent reviewer, with complimentary lived experience in a different professional sport or MCT work sector, may have further strengthened the reflexive interpretation of responses.

The psychological skills necessary to perform in professional sports and MCTs share key features justifying the approach of this investigation. However, the contextual demands, magnitude of stressors and irreversibility of outcomes can vary substantially within, and between, professional sports and MCTs. Therefore, investigating mindsets through more refined and contextually specific subcategories of professional sports and MCTs may be beneficial.

The statistical methods used in this study, while appropriate for its exploratory aims, do not permit inferential conclusions or a comprehensive evaluation of the instrument's psychometric properties. Future research should consider more advanced approaches, such as Mann–Whitney U or Kruskal–Wallis tests to examine sub-group differences. Although assessing test-retest reliability using Spearman's Rho and Quadratic Weighted Kappa evaluates temporal stability and ordinal agreement it does not assess the underlying psychometric structure of the instrument. More sophisticated approaches such as Item Response Theory and/or Confirmatory Factor Analysis would provide a more rigorous assessment of construct validity, item-level discrimination and measurement precision.

A further interpretive limitation concerns the theoretical integration adopted in this study. The working definition of ‘mindset’ employed deliberately spans constructs – including growth mindset, stress mindset, expectancy effects and placebo-related mechanisms – that originate from distinct empirical traditions. Whilst a shared-belief architecture provides a principled rationale for this integration in the context of exploratory, practitioner-focused inquiry, the boundaries between these constructs should not be treated as dissolved.

Most respondents identified themselves as male which limits the generalisability of these findings for females working in professional sport or MCTs. Additionally, there were fewer respondents from emergency medicine and firefighting which, again, limits the generalisability of these findings in these work sectors. In both instances, future research may wish to specifically investigate the beliefs and perceptions of female Performers and PSS and/or specific MCTs.

## Data Availability

The datasets overviewed in this investigation are not readily available for dissemination as the consent process agreed to by participants did not specifically ask their permission to openly share their information. Therefore, to share any data, we would need to seek updated consent from each participant. Requests to access the datasets should be directed to David Gray, gray.david@ul.ie.
