# Supplementary material for: Mindsets impact training adaptation and high-pressure performance: beliefs and perceptions of professionals working in elite sport and mission critical teams
Source: Front Sports Act Living. 2026 Jun 22;8:1827074. doi: 10.3389/fspor.2026.1827074 (PMC13333680; doi:10.3389/fspor.2026.1827074)
Supplement: Supplementary file 1 [file Supplementaryfile1.docx]

Supplementary Information

Mindsets Impact Training Adaptation and High-Pressure Performance: Beliefs and Perceptions of Professionals Working in Elite Sport and Mission Critical Teams

David Gray and John Kiely

**Appendix Supplementary Information Page**

1. Survey Inclusion Criteria 2
2. Survey Recruitment Material 3-4
3. Copy of Survey 5-42
4. Test-Retest Reliability 43-44

**Appendix 1: Survey Inclusion Criteria**

The set inclusion criteria was:

- Aged 18+ years.
- English is either: a) your native language b) a language you speak fluently or c) the primary language used in your work setting.
- Currently work, or within the last 10 years, worked in one of the following work settings: a) professional sport b) specialist police unit c) specialist military unit d) firefighting e) emergency medicine f) surgery g) other first-responder h) other (please specify)

Participants were required to self-classify their primary professional role(s) as either a Performer or Performance Support Staff (PSS):

- Performer role: which includes (but not limited to) athlete, surgeon, emergency medical response team member, special forces operator, tactical police operator, firefighter, pilot.
- PSS role: which includes (but not limited to) head coach, coach, instructor, tactical commander, strength & conditioning coach, physiotherapist, performance director, performance psychologist, mental skill trainer.

Participants with dual roles and/or uncertainty over which category of respondent to select were advised to choose the option that aligned to the major component of their work over the previous 2 years.

**Appendix 2: Survey Recruitment Material**

**
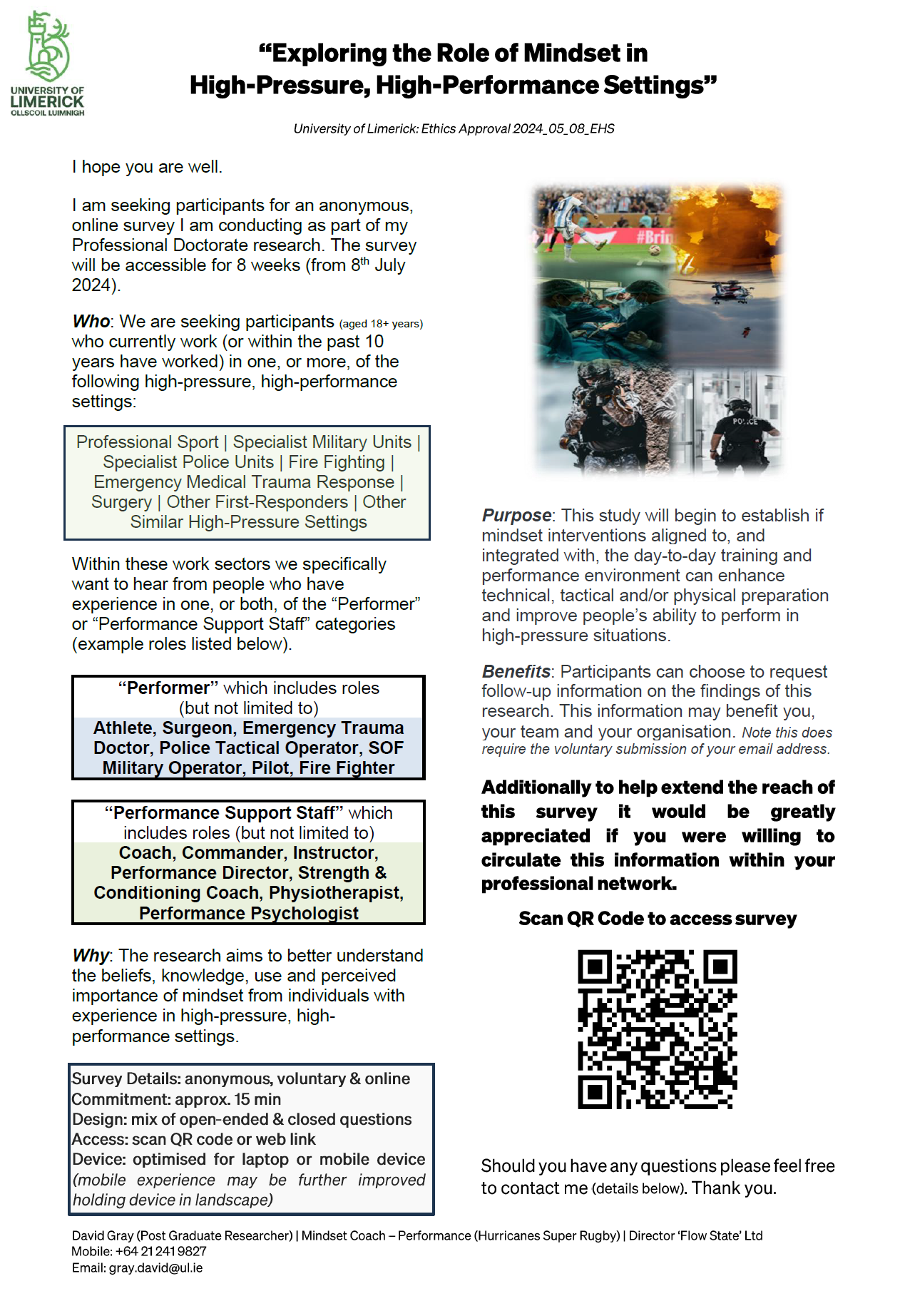
**

**
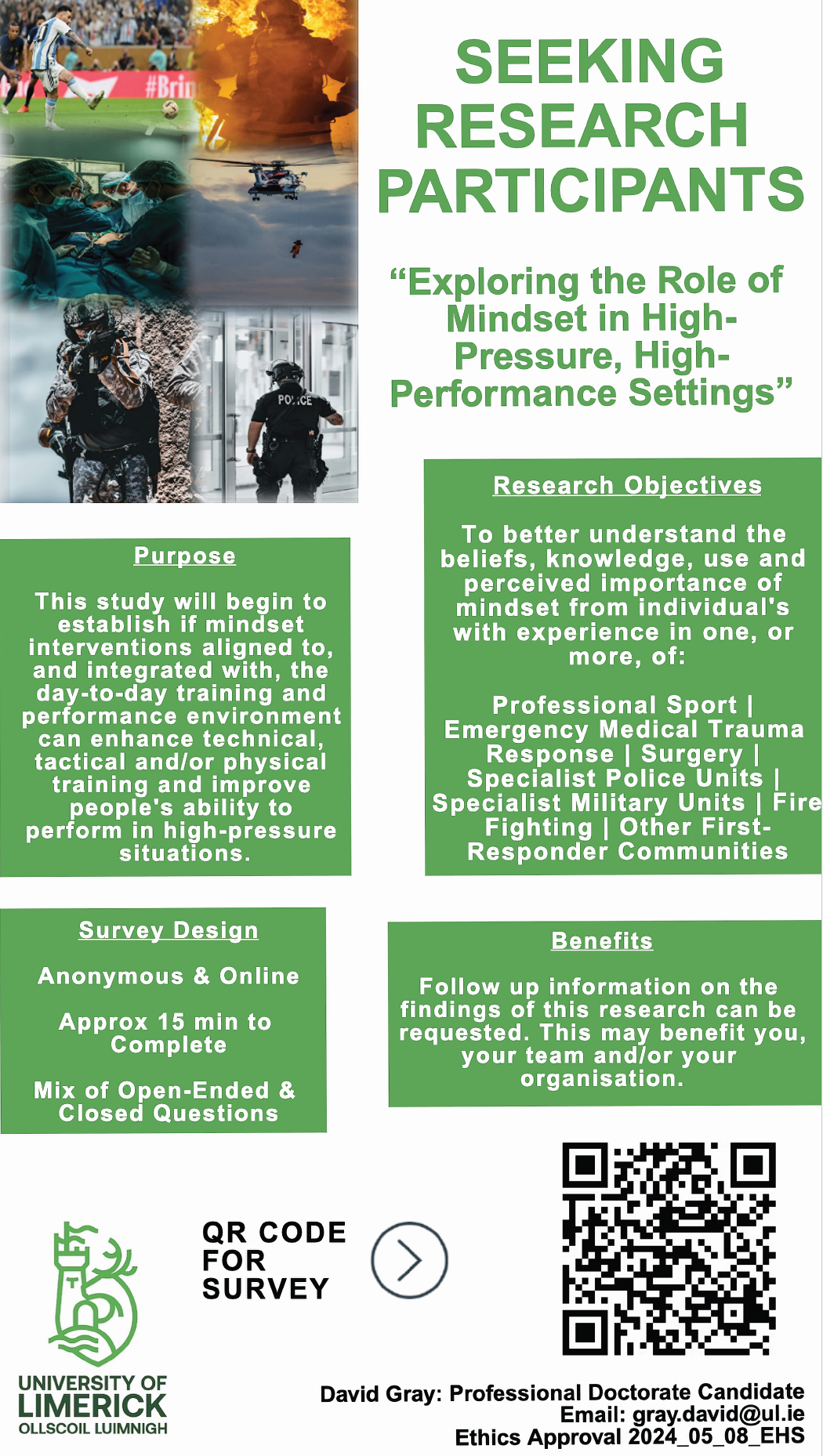
**

**Appendix 3: Copy of Survey**

Standard: Introduction (1 Question)

Standard: Information Sheet (1 Question)

Block: Survey (60 Questions)

Branch: New Branch

If

If To customise the survey please select the category of respondent that best reflects your current... "Performer" (e.g. athlete, surgeon, tactical operator, SF soldier, pilot, firefighter, trauma response doctor) Is Selected

Branch: New Branch

If

If To customise the survey please select the category of respondent that best reflects your current... "Performance Support Staff" (e.g. coach, commander, instructor, performance psychologist, performance director, strength & conditioning coach, physiotherapist, mental skills trainer) Is Selected

Branch: New Branch

If

If In your professional career have you ever received mindset-specific education? No Is Selected

And In your professional career have you ever received mindset-specific education? Unsure Is Selected

Branch: New Branch

If

If In your professional career have you ever received mindset-specific education? Yes Is Selected

| Page Break |  |
| --- | --- |

Start of Block: Introduction

Q107

 **"Exploring the Role of Mindset in High-Pressure,**
**High-Performance Settings"**
  
Thank you for expressing an interest in completing this survey which is being conducted by David Gray
(a professional doctorate candidate at the University of Limerick
and performance coach with 20 years' experience in professional sport)
and Dr John Kiely
Associate Professor
in Human Performance and Innovation
(University of Limerick).


David can be reached by email at gray.david@ul.ie

End of Block: Introduction

Start of Block: Information Sheet

Q131
**PARTICIPANT INFORMATION SHEET**
  
**Exploring the role of Mindset in High-Pressure, High-Performance Settings.**
 As part of my Doctorate Research, at the University of Limerick, I am carrying out a study exploring the role of mindset in high-pressure, high-performance settings. This participant information sheet will inform you what the study is about. This research is being conducted by the David Gray (Doctoral candidate) and Dr. John Kiely. We appreciate your interest in participating in this online survey.

 **What is the study about**? Our aim is to gather information on the beliefs, knowledge, use and perceived importance of mindset from people with experience in high-pressure, high-performance settings.

 **What is the participation criteria**? We are interested in individuals who **currently work** (**or within the last 10 years have worked**) in professional sport, emergency medicine, surgery, firefighting, specialist police units, specialist military units, other first-responder communities (or similar high-pressure, high-performance environments).
   
 We are specifically looking to recruit:

 Individuals who have/had a "**Performer**" role which includes (but not limited to) **athlete, surgeon, emergency medical response team, surgeon, special forces operator, tactical police officer, firefighter, pilot.**

 **OR**

 Individuals who have/had a "**Performance Support Staff**" role which includes (but not limited to) **head coach, coach, instructor, commander, strength & conditioning coach, physiotherapist, performance director, performance psychologist, mental skills trainer**.

 **What will I have to do**? You will complete a one-off anonymous online survey. The survey contains a mix of open-ended and closed questions and takes approximately 15 minutes to complete. There will be questions relating to:
   Demographic information Experience of high-pressure events How you utilise mindset (if at all) in high-pressure situations Your beliefs, knowledge and perspectives on the usefulness of mindset Experience of any mindset-specific education
 The survey is voluntary, and all survey responses will be completely anonymised.

 **Who are the researchers**? This research project is being conducted by David Gray, an applied practitioner with 20 years of coaching experience in professional sport, and a Professional Doctorate student, alongside Dr. John Kiely an Associate Professor in Performance & Innovation at the University of Limerick.

 **What are the benefits**? Findings from this study will allow us to determine the current level of knowledge and understanding of mindset, and, whether mindset is perceived as being useful and important in high-pressure, high-performance environments. Additionally, this study will begin to establish if mindset interventions aligned to, and integrated with, the day-to-day training and performance environment can enhance technical, tactical and/or physical preparation and improve people's ability to perform in high-pressure situations.

 ***Should you, or your team/organisation, be interested there is a voluntary option to request follow up information on the findings of this survey and our future research.***

 **What are the risks**? You do not have to answer any question you do not wish to. Recalling your experiences of high-pressure situations may remind you of events that caused physical and/or psychological harm to yourself or others. Any potential distress is unlikely to exceed everyday experiences.

 **What if I do not want to take part**? Participation in this study is voluntary and you can choose not to take part or to stop your involvement in this study at any time.

 **What happens to the information**? The survey information is collected online, using the University approved Qualtrics survey software. Once collected, all data will be kept private and stored securely and safely on the University of Limerick’s approved OneDrive cloud and on the research team’s password protected computers. Your name will not appear on any information as surveys will be completed using an anonymous link. Your data will only be accessible to the research team. By participating in this survey, you consent to your data being collected and used for research purposes. The information gathered in the study will be kept for a maximum of seven years. After this time, it will be destroyed. Further information on how your data is securely stored is available in the Research Privacy Notice which is accessible through the following link:
 https://ulcampus-my.sharepoint.com/:b:/g/personal/23291958_studentmail_ul_ie/Ea6BCZZSCgxEkpo51qp8BhIBwk2kJ40rfkyWTNrJ6JRO9Q?e=SqCc7g

 **Who else is taking part**? This research focuses on professionals **currently working (or within the last 10 years have worked)** in one of the listed high-pressure, high-performance settings. Participants will either be:

 Individuals who have/had a "**Performer**" role which includes (but not limited to) athlete, surgeon, emergency medical response team, surgeon, special forces operator, tactical police officer, firefighter, pilot

 **OR**

 Individuals who have/had a "**Performance Support Staff**" role which includes (but not limited to) head coach, coach, instructor, commander, strength & conditioning coach, physiotherapist, performance director, performance psychologist, mental skills trainer

 **What if something goes wrong**? The online survey is voluntary and you can exit the survey at any time.

 **What happens at the end of the study**? At the end of the study the information will be used in a doctoral thesis and, potentially, published in academic publications. The information will be completely anonymous, you cannot be identified and no names (or identifying information) will appear in any reporting of results.

 **What if I have more questions or do not understand something**? If you have any questions about the study you may contact either of the researchers. It is important that you feel that all your questions have been answered.

 **What happens if I change my mind during the study**? At any stage should you feel that you want to stop taking part in the study, you are free to stop and withdraw. There are no consequences for changing your mind about being in the study.

 Contact name and email of Project Investigators:
 Principal Investigator Dr. John Kiely
 Faculty Member, PESS Dept, University of Limerick
 Email: john.kiely@ul.ie

 Other investigator
 David Gray
 Professional Doctorate Candidate, PESS Department, University of Limerick.
 Email: gray.david@ul.ie

 Thank you for taking the time to read this. We would be grateful if you would consider participating in this study.

Yours sincerely,

  Dr. John Kiely
  David Gray (Doctoral candidate)

 This research study has received Ethics approval from the Education and Health Sciences Research Ethics Committee (approval number 2024_05_08_EHS). If you have any concerns about this study and wish to contact someone independent you may contact: Chair Education and Health Sciences Research Ethics Committee EHS Faculty Office University of Limerick (Ireland) Tel (061) 234101

End of Block: Information Sheet

Start of Block: Survey

Q149
**Participant Eligibility**

Q102 Please confirm you provide your voluntary consent to participate in this survey.

- Yes (1)
- No (2)

Skip To: End of Survey If Please confirm you provide your voluntary consent to participate in this survey. = No

Q147 Please confirm you are 18+ years of age.

- Yes (1)
- No (2)

Skip To: End of Survey If Please confirm you are 18+ years of age. = No

Q148 Please confirm English is either:

 1) your native language **OR**
 2) you are fluent in English **OR**
 3) English is the primary language used in your high-pressure, high-performance work setting.

- Yes (1)
- No (2)

Skip To: End of Survey If Please confirm English is either: 1) your native language OR 2) you are fluent in English OR 3) E... = No

| Page Break |  |
| --- | --- |

Q143
**Demographic Information**

Q1 Please select **all** the work sectors that you either:

 **Cu**rrently work in
 OR
 **Last** worked in (if not currently working in a high-pressure, high-performance setting)

 *(Note: This question only refers to your current (or last) work experience. You will be asked about any other relevant experience later in the survey)*

- Professional Sport (please specify below sport, role, full-time/part-time and highest level of competition) (1) __________________________________________________
- Specialist Police Units (please specify below specialist role, full-time/part-time & unit/team) (2) __________________________________________________
- Specialist Military Units (please specify below specialist role, full-time/part-time & unit/team) (3) __________________________________________________
- Fire Fighting (please specify below specialist role, full-time/part-time & unit/team) (4) __________________________________________________
- Emergency Medicine (please specify below specialist role, full-time/part-time & unit/team) (5) __________________________________________________
- Surgery (please specify below specialist role, full-time/part-time & unit/department) (6) __________________________________________________
- Other First-Responder (please specify below work sector, specialist role, full-time/part-time & unit/team/department) (7) __________________________________________________
- Other (please specify below work sector, specialist role, full-time/part-time & unit/team/department) (8) __________________________________________________

| Page Break |  |
| --- | --- |

Q5 Are you **currently** working in this role(s)?

- Yes (1)
- No (2)

Display This Question:

If Are you currently working in this role(s)? = No

Q39 Please confirm you worked in these role(s) **within the last 10 years** (since 2014)

- Yes (1)
- No (please note selecting this option will end your survey as you do not meet the participation criteria) (2)

Skip To: End of Survey If Please confirm you worked in these role(s) within the last 10 years (since 2014) = No (please note selecting this option will end your survey as you do not meet the participation criteria)

Q36 Number of years experience in your **current** (**or last**) role?

- 0-1 year (1)
- 2-4 years (2)
- 5-10 years (3)
- 11-15 year (4)
- 16-20 years (5)
- 20+ years (6)

| Page Break |  |
| --- | --- |

Q37 Have you gained any **additional experience** in high-pressure, high-performance settings? *(e.g. a current professional coach may have prior experience as a professional athlete, a current firefighter may have previous experience working in military special operations, an emergency medicine professional may have previous experience in search & rescue before they transitioned to medicine)*

- Yes (1)
- No (2)

Display This Question:

If Have you gained any additional experience in high-pressure, high-performance settings? (e.g. a cu... = Yes

Q112 Select **all** the work sectors where you have gained additional experience?

- Professional Sport (please specify # years, sport, full-time/part-time, role and highest level of competition) (1) __________________________________________________
- Specialist Police Units (please specify # years, specialist role, full-time/part-time & unit/team) (2) __________________________________________________
- Specialist Military Units (please specify # years, specialist role, full-time/part-time & unit/team) (3) __________________________________________________
- Fire Fighting (please specify # years, specialist role, full-time/part-time & unit/team) (4) __________________________________________________
- Emergency Medicine (please specify # years, specialist role, full-time/part-time & unit/team) (5) __________________________________________________
- Surgery (please specify # years, specialist role, full-time/part-time & unit/department) (6) __________________________________________________
- Other First-Responder (please specify work sector, # years, specialist role, full-time/part-time & unit/team/department) (7) __________________________________________________
- Other (please specify work sector, # years, specialist role, full-time/part-time & unit/team/department) (8) __________________________________________________

Display This Question:

If Have you gained any additional experience in high-pressure, high-performance settings? (e.g. a cu... = Yes

Q7 **In total**, across all your different roles (including your current or last role), how much experience do you have in high-pressure, high-performance environments?

- 0-5 years (1)
- 6-10 years (2)
- 11-15 years (3)
- 16-20 years (4)
- 21-25 years (5)
- 26-30 years (6)
- 30+ years (7)

Q6 What is your age?

- 18-25 years (1)
- 26-35 years (2)
- 36-45 years (3)
- 46-55 years (4)
- 56+ years (5)

Q8 Nationality

________________________________________________________________

| Page Break |  |
| --- | --- |

Q9 Gender

- Male (1)
- Female (2)
- Non-binary / third gender (3)
- Prefer not to say (4)

Q10 To customise the survey please select the category of respondent that best reflects your current (or last) role. *(note: if you could select both "performer" and "performance support staff" please select the option which aligns to the major component of your work over the last 2 years).*

- "Performer" (e.g. athlete, surgeon, tactical operator, SF soldier, pilot, firefighter, trauma response doctor) (1)
- "Performance Support Staff" (e.g. coach, commander, instructor, performance psychologist, performance director, strength & conditioning coach, physiotherapist, mental skills trainer) (2)
- Unsure (please note selecting this option will end your survey) (3)

Skip To: End of Survey If To customise the survey please select the category of respondent that best reflects your current... = Unsure (please note selecting this option will end your survey)

| Page Break |  |
| --- | --- |

Q34
**The following section primarily contains open-ended questions.**


**When answering these questions we encourage you to openly share your ideas, thinking and beliefs.**


**Your personal insights have tremendous value to this research.**


**There are no right or wrong answers to these questions.**
 
**Please respond N/A to any questions that are not applicable to you.**

Q145 I consider myself to be aware of the concept of '**Mindset**'.

- Strongly disagree (1)
- Disagree (2)
- Somewhat disagree (3)
- Neither agree or disagree (4)
- Somewhat agree (5)
- Agree (6)
- Strongly agree (7)
- Unsure (8)

Q12 Briefly describe what the term '**mindset**' means to you? *(please respond in 3-4 sentences max)*

________________________________________________________________

________________________________________________________________

________________________________________________________________

________________________________________________________________

________________________________________________________________

Display This Question:

If To customise the survey please select the category of respondent that best reflects your current... = "Performer" (e.g. athlete, surgeon, tactical operator, SF soldier, pilot, firefighter, trauma response doctor)

Q13 Describe a typical **high-pressure** situation that you regularly encounter in your role? *(please add as much detail as you feel necessary)*

________________________________________________________________

________________________________________________________________

________________________________________________________________

________________________________________________________________

________________________________________________________________

Display This Question:

If To customise the survey please select the category of respondent that best reflects your current... = "Performer" (e.g. athlete, surgeon, tactical operator, SF soldier, pilot, firefighter, trauma response doctor)

Q108 What tools, strategies and/or techniques (if any) do you utilise to optimise **mindset** leading into, and during, **high-pressure** situations? *(list up to 4 key tools/strategies/techniques & the reason(s) this benefits your mindset)*

- Mindset Strategy 1 (1) __________________________________________________
- Mindset Strategy 2 (2) __________________________________________________
- Mindset Strategy 3 (3) __________________________________________________
- Mindset Strategy 4 (4) __________________________________________________

Display This Question:

If To customise the survey please select the category of respondent that best reflects your current... = "Performer" (e.g. athlete, surgeon, tactical operator, SF soldier, pilot, firefighter, trauma response doctor)

Q15 Reflecting on your performance during **high-pressure** situations, how has **mindset** been helpful to your performance, if at all? *(please add as much detail as you feel necessary)*

________________________________________________________________

________________________________________________________________

________________________________________________________________

________________________________________________________________

________________________________________________________________

Display This Question:

If To customise the survey please select the category of respondent that best reflects your current... = "Performer" (e.g. athlete, surgeon, tactical operator, SF soldier, pilot, firefighter, trauma response doctor)

Q119 Does **mindset** impact how people respond, and adapt, to technical, tactical and/or physical training?

- Yes (1)
- No (2)
- Unsure (3)

| Page Break |  |
| --- | --- |

Display This Question:

If Does mindset impact how people respond, and adapt, to technical, tactical and/or physical training? = Yes

And To customise the survey please select the category of respondent that best reflects your current... = "Performer" (e.g. athlete, surgeon, tactical operator, SF soldier, pilot, firefighter, trauma response doctor)

Q113 In what specific aspects of technical, tactical and/or physical training does **mindset** have the most impact on training response / adaptation?  *(List up to 3 areas and a brief explanation on why you believe this)*

- Example 1 (1) __________________________________________________
- Example 2 (2) __________________________________________________
- Example 3 (3) __________________________________________________

Display This Question:

If To customise the survey please select the category of respondent that best reflects your current... = "Performance Support Staff" (e.g. coach, commander, instructor, performance psychologist, performance director, strength & conditioning coach, physiotherapist, mental skills trainer)

Q47 Briefly describe *(in 4-5 sentences max)* the typical coaching/training/teaching/instructing you deliver in your 'performance support' role?

________________________________________________________________

________________________________________________________________

________________________________________________________________

________________________________________________________________

________________________________________________________________

Display This Question:

If To customise the survey please select the category of respondent that best reflects your current... = "Performance Support Staff" (e.g. coach, commander, instructor, performance psychologist, performance director, strength & conditioning coach, physiotherapist, mental skills trainer)

Q116 What tools, strategies, techniques, models/approaches (if any) do you utilise to optimise the **mindset**of the "performers" you work with? *(list up to 4 key tools/strategies/techniques & the reasons you feel these optimise mindset)*

- Mindset Strategy 1 (1) __________________________________________________
- Mindset Strategy 2 (2) __________________________________________________
- Mindset Strategy 3 (3) __________________________________________________
- Mindset Strategy 4 (4) __________________________________________________

Display This Question:

If To customise the survey please select the category of respondent that best reflects your current... = "Performance Support Staff" (e.g. coach, commander, instructor, performance psychologist, performance director, strength & conditioning coach, physiotherapist, mental skills trainer)

Q49 In your professional opinion, how is **mindset** beneficial to performance in **high-pressure** situations, if at all? *(please add as much detail as you feel necessary)*

________________________________________________________________

________________________________________________________________

________________________________________________________________

________________________________________________________________

________________________________________________________________

Display This Question:

If To customise the survey please select the category of respondent that best reflects your current... = "Performance Support Staff" (e.g. coach, commander, instructor, performance psychologist, performance director, strength & conditioning coach, physiotherapist, mental skills trainer)

Q135 Does **mindset** impact how people respond, and adapt, to technical, tactical and/or physical training?

- Yes (1)
- No (2)
- Unsure (3)

Display This Question:

If Does mindset impact how people respond, and adapt, to technical, tactical and/or physical training? = Yes

And To customise the survey please select the category of respondent that best reflects your current... = "Performance Support Staff" (e.g. coach, commander, instructor, performance psychologist, performance director, strength & conditioning coach, physiotherapist, mental skills trainer)

Q125 In what specific aspects of technical, tactical and/or physical training does **mindset** most impact training response / adaptation?  *(List up to 3 areas and a brief explanation on why you believe this)*

- Example 1 (1) __________________________________________________
- Example 2 (2) __________________________________________________
- Example 3 (3) __________________________________________________

Display This Question:

If Does mindset impact how people respond, and adapt, to technical, tactical and/or physical training? = Yes

And To customise the survey please select the category of respondent that best reflects your current... = "Performance Support Staff" (e.g. coach, commander, instructor, performance psychologist, performance director, strength & conditioning coach, physiotherapist, mental skills trainer)

Q126 In your opinion how does someone's **mindset** have an impact on their response and adaptation to training?

________________________________________________________________

________________________________________________________________

________________________________________________________________

________________________________________________________________

________________________________________________________________

Display This Question:

If To customise the survey please select the category of respondent that best reflects your current... = "Performance Support Staff" (e.g. coach, commander, instructor, performance psychologist, performance director, strength & conditioning coach, physiotherapist, mental skills trainer)

Q49 In specific relation to **mindset** what, if anything, could be done to further improve the coaching/training/teaching/instructing being delivered in **high-pressure, high-performance** settings? *(please add as much detail as you feel necessary)*

________________________________________________________________

________________________________________________________________

________________________________________________________________

________________________________________________________________

________________________________________________________________

Q118 Could you take a moment to reflect on the **performance environment** you are involved in day to day. What factors, if any, do you perceive as having the most impact on **mindset**? *(list up to 3 factors and whether these have positive or negative effects)*

- Key Factor 1 (1) __________________________________________________
- Key Factor 2 (2) __________________________________________________
- Key Factor 3 (3) __________________________________________________

| Page Break |  |
| --- | --- |

Q62
There are several definitions of the term '**mindset**'
currently available in the published literature. 
 
To objectify responses across the remainder of this survey
could you base the rest of your responses on
this working definition of mindset:

 ***"Mindset refers to our core beliefs which act as lenses that guide our future emotions, thinking, behaviours and actions"***

Q110 How important has your **mindset** been in helping you reach your high-level of performance?

|  | Not at all important | Very limited importance | Slightly important | Moderately important | Important | Very important | Critical | Unsure |
| --- | --- | --- | --- | --- | --- | --- | --- | --- |

|  | 1 | 2 | 3 | 4 | 5 | 6 | 7 |
| --- | --- | --- | --- | --- | --- | --- | --- |

| Drag & Drop Slider () | 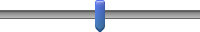 |
| --- | --- |

Q111 What specific characteristics, related to your **mindset,** have helped you reach your high-level of performance? *(list up to 3)*

- Mindset characteristic 1 (1) __________________________________________________
- Mindset characteristic 2 (2) __________________________________________________
- Mindset characteristic 3 (3) __________________________________________________

Q35 Based on your typical workplace interactions *(e.g. conversations, debriefs, operational reviews)* how important is **mindset** to other members of your team/organisation?

|  | Not at all important | Very limited importance | Slightly important | Moderately important | Important | Very important | Critical | Unsure |
| --- | --- | --- | --- | --- | --- | --- | --- | --- |

|  | 1 | 2 | 3 | 4 | 5 | 6 | 7 |
| --- | --- | --- | --- | --- | --- | --- | --- |

| Drag & Drop Slider () | 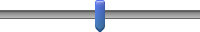 |
| --- | --- |

| Page Break |  |
| --- | --- |

Q140
*This is a reminder message of the working definition of mindset.*

 ***"Mindset refers to our core beliefs which act as lenses that guide our future emotions, thinking, behaviours and actions"***

Q24 **Mindsets** trigger physiological, psychological and behavioural responses that can either be **adaptive** (beneficial) or **maladaptive** (damaging) to both training and performance outcomes.

- Strongly disagree (1)
- Disagree (2)
- Somewhat disagree (3)
- Neither agree or disagree (4)
- Somewhat agree (5)
- Agree (6)
- Strongly agree (7)
- Unsure (8)

Q142 Based on the specific demands of a task, or situation, I will deliberately transition/switch between different **Mindsets** to optimise my performance.

- Strongly disagree (1)
- Disagree (2)
- Somewhat disagree (3)
- Neither agree or disagree (4)
- Somewhat agree (5)
- Agree (6)
- Strongly agree (7)
- Unsure (8)

Display This Question:

If To customise the survey please select the category of respondent that best reflects your current... = "Performer" (e.g. athlete, surgeon, tactical operator, SF soldier, pilot, firefighter, trauma response doctor)

Q72 My deep understanding of **mindset** optimises my adaptation to training and my performance during high-pressure situations.

- Strongly disagree (1)
- Disagree (2)
- Somewhat disagree (3)
- Neither agree or disagree (4)
- Somewhat agree (5)
- Agree (6)
- Strongly agree (7)
- Unsure (8)

Display This Question:

If To customise the survey please select the category of respondent that best reflects your current... = "Performance Support Staff" (e.g. coach, commander, instructor, performance psychologist, performance director, strength & conditioning coach, physiotherapist, mental skills trainer)

Q73 I have a deep understanding and knowledge of **mindset** which optimises the coaching/training/teaching/instructing I prescribe.

- Strongly disagree (1)
- Disagree (2)
- Somewhat disagree (3)
- Neither agree or disagree (4)
- Somewhat agree (5)
- Agree (6)
- Strongly agree (7)
- Unsure (8)

| Page Break |  |
| --- | --- |

Q151
*This is a reminder message of the working definition of mindset.*

 ***"Mindset refers to our core beliefs which act as lenses that guide our future emotions, thinking, behaviours and actions"***

Q75 **Mindsets** can positively impact an individual's:

|  | Strongly disagree (1) | Disagree (2) | Somewhat disagree (3) | Neither agree or disagree (4) | Somewhat agree (5) | Agree (6) | Strongly agree (7) | Unsure (8) |
| --- | --- | --- | --- | --- | --- | --- | --- | --- |
| Physiological adaptation to training (1) |  |  |  |  |  |  |  |  |
| Perception of our abilities & intelligence (2) |  |  |  |  |  |  |  |  |
| Goal-oriented behaviours (3) |  |  |  |  |  |  |  |  |
| Motivation (4) |  |  |  |  |  |  |  |  |
| Visual scanning & perception (5) |  |  |  |  |  |  |  |  |
| Decision-making (6) |  |  |  |  |  |  |  |  |
| Focus & attention (7) |  |  |  |  |  |  |  |  |
| Technical skill execution (8) |  |  |  |  |  |  |  |  |
| Physiological & psychological response to high-pressure situations (9) |  |  |  |  |  |  |  |  |

Q33 Are **mindsets** more **fixed** (cannot be changed) or more **malleable** (can be changed)?

|  | Fully fixed | Predominantly fixed | Somewhat fixed | Equal balance between fixed and malleable | Somewhat malleable | Predominantly malleable | Fully malleable | Unsure |
| --- | --- | --- | --- | --- | --- | --- | --- | --- |

|  | 1 | 2 | 3 | 4 | 5 | 6 | 7 |
| --- | --- | --- | --- | --- | --- | --- | --- |

| Drag & Drop Slider () | 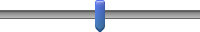 |
| --- | --- |

Q132 Is **mindset** important in the final few minutes **BEFORE** a high-pressure situation?

- Yes (please briefly explain why you think this) (1) __________________________________________________
- No (please briefly explain why you think this) (2) __________________________________________________
- Unsure (please briefly explain why you think this) (3) __________________________________________________

Q133 **DURING** high-pressure situations is **mindset** important in optimising:

|  | Not important at all (1) | Very limited importance (2) | Slightly important (3) | Moderately important (4) | Important (5) | Very important (6) | Critical (7) | Unsure (8) |
| --- | --- | --- | --- | --- | --- | --- | --- | --- |
| Technical Skill Execution (1) |  |  |  |  |  |  |  |  |
| Tactical Performance (e.g. decision making, problem solving) (2) |  |  |  |  |  |  |  |  |
| Physical Performance (3) |  |  |  |  |  |  |  |  |

| Page Break |  |
| --- | --- |

Q153
*This is a reminder message of the working definition of mindset.*

 ***"Mindset refers to our core beliefs which act as lenses that guide our future emotions, thinking, behaviours and actions"***

Q28 In your professional career have you ever received **mindset-specific** education?

- Yes (1)
- No (2)
- Unsure (3)

Display This Question:

If In your professional career have you ever received mindset-specific education? = No

Or In your professional career have you ever received mindset-specific education? = Unsure

Q109 Would accessing **mindset-specific** education have helped you perform more effectively in your role?

- Yes (1)
- No (2)
- Unsure (3)

Display This Question:

If In your professional career have you ever received mindset-specific education? = Yes

Q98 Could you briefly describe the format, and frequency, of the **mindset-specific** education you received.

________________________________________________________________

________________________________________________________________

________________________________________________________________

________________________________________________________________

________________________________________________________________

| Page Break |  |
| --- | --- |

Q154
*This is a reminder message of the working definition of mindset.*

 ***"Mindset refers to our core beliefs which act as lenses that guide our future emotions, thinking, behaviours and actions"***

Display This Question:

If In your professional career have you ever received mindset-specific education? = Yes

And To customise the survey please select the category of respondent that best reflects your current... = "Performer" (e.g. athlete, surgeon, tactical operator, SF soldier, pilot, firefighter, trauma response doctor)

Q134 How important was this **mindset-specific** education in optimising your adaptation, and response, to:

|  | Not important at all (1) | Very limited importance (2) | Slightly important (3) | Moderately important (4) | Important (5) | Very important (6) | Critical (7) | Unsure (8) |
| --- | --- | --- | --- | --- | --- | --- | --- | --- |
| Technical Skill Training (1) |  |  |  |  |  |  |  |  |
| Tactical Training (e.g. decision making, problem solving) (2) |  |  |  |  |  |  |  |  |
| Physical Training (3) |  |  |  |  |  |  |  |  |

Display This Question:

If To customise the survey please select the category of respondent that best reflects your current... = "Performer" (e.g. athlete, surgeon, tactical operator, SF soldier, pilot, firefighter, trauma response doctor)

And In your professional career have you ever received mindset-specific education? = Yes

Q100 How important was this **mindset-specific** education in optimising your performance during **high-pressure** situations?

|  | Not at all important | Very little importance | Slightly important | Moderately important | Important | Very important | Critical | Unsure |
| --- | --- | --- | --- | --- | --- | --- | --- | --- |

|  | 1 | 2 | 3 | 4 | 5 | 6 | 7 |
| --- | --- | --- | --- | --- | --- | --- | --- |

| Drag & Drop Slider () | 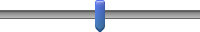 |
| --- | --- |

Display This Question:

If To customise the survey please select the category of respondent that best reflects your current... = "Performance Support Staff" (e.g. coach, commander, instructor, performance psychologist, performance director, strength & conditioning coach, physiotherapist, mental skills trainer)

And In your professional career have you ever received mindset-specific education? = Yes

Q99 How important was this **mindset-specific** education in improving your coaching/teaching/training/instructing?

|  | Not at all important | Very little importance | Slightly important | Moderately important | Important | Very important | Critical | Unsure |
| --- | --- | --- | --- | --- | --- | --- | --- | --- |

|  | 1 | 2 | 3 | 4 | 5 | 6 | 7 |
| --- | --- | --- | --- | --- | --- | --- | --- |

| Drag & Drop Slider () | 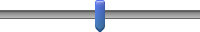 |
| --- | --- |

| Page Break |  |
| --- | --- |

Q152
*This is a reminder message of the working definition of mindset.*

 ***"Mindset refers to our core beliefs which act as lenses that guide our future emotions, thinking, behaviours and actions"***

Q103 Would a **mindset-education resource** specifically designed to support people working in high-pressure, high-performance environments be of interest to you?

- Yes, definitely (1)
- Yes, highly likely (2)
- Yes, possibly (3)
- Unsure, would need to know more about it (4)
- No (5)

Display This Question:

If Would a mindset-education resource specifically designed to support people working in high-pressu... = Yes, definitely

Or Would a mindset-education resource specifically designed to support people working in high-pressu... = Yes, highly likely

Or Would a mindset-education resource specifically designed to support people working in high-pressu... = Yes, possibly

Or Would a mindset-education resource specifically designed to support people working in high-pressu... = Unsure, would need to know more about it

| 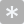 |
| --- |

Q104 In what format would a **mindset-education resource** appeal the most? *(please drag & drop up to 2 preferences)*

| Ranked Order of Preference |
| --- |
| ______ Online delivery (e.g. self-paced course, e-learning modules, webinars) (1) |
| ______ In-person delivery (2) |
| ______ Audio format (e.g. podcast, audio book) (3) |
| ______ Written text (e.g. book, short articles, research papers) (4) |
| ______ Other (please specifiy) (5) |

| Page Break |  |
| --- | --- |

Q106 Based on your own learnings and experiences if you could share one piece of **mindset-related** advice, that would positively impact someone's ability to **perform in high-pressure situations**, what would it be? *(please add as much detail as you wish)*

________________________________________________________________

________________________________________________________________

________________________________________________________________

________________________________________________________________

________________________________________________________________

| Page Break |  |
| --- | --- |

Q50 Are you interested in learning about the results of this study and/or future research conducted by the research team?

- Yes (please add your name and email address below) (1) __________________________________________________
- No (2)

Q121 Would you be willing to help us assess the reliability of this survey by re-taking the survey in 2 weeks? *(a separate survey link will be sent to you by email)*

- Yes (please add your name & email address below unless provided earlier) (1) __________________________________________________
- No (2)

**Appendix 4: Test-Retest Reliability**

Test-retest level of agreement and correlation

| **Question** | **Performer**  **n =** | **PSS**  **n =** | κ_q_  **(95% CI)** | **p-value**  **(**κ_q_**)** | **Spearman’s rho** | **p-value (rho)** |
| --- | --- | --- | --- | --- | --- | --- |
| I consider myself to be aware of the concept of mindset | 10 | 13 | 0.65 (0.442-0.859) | .001 | 0.711** | <.001 |
| How important has your mindset been in helping you reach your high level of performance? | 10 | 13 | 0.145 (-0.159-0.449) | .344 | 0.206 | .347 |
| Based on your typical workplace interactions (e.g. conversations, debriefs, operational reviews) how important is mindset to other members of your team/organisation? | 9 | 11 | 0.429 (0.076-0.781) | .052 | 0.419 | .066 |
| Mindsets trigger physiological, psychological and behavioural responses that can be adaptive (beneficial) or maladaptive (detrimental) to both training and performance outcomes | 10 | 13 | 0.087 (-0.29-0.464) | .658 | 0.145 | .509 |
| Based on the specific demands of a task, or situation, I will deliberately transition/switch between different mindsets to optimise my performance | 10 | 13 | 0.534 (0.094-0.974) | .002 | 0.432* | .040* |
| My deep understanding of mindset optimises my adaptation to training and performance during high-pressure situations | 10 | n/a | -0.25 (-0.831-0.331) | .429 | -0.25 | .486 |
| I have a deep understanding and knowledge of mindset which optimises the coaching / training / teaching / instructing I prescribe | n/a | 13 | 0.585 (0.298-0.872) | .17 | 0.668* | .13 |
| Mindsets can positively impact an individual’s: physiological adaptation to training | 10 | 13 | 0.589 (0.229-0.949) | .005 | 0.589** | .003 |
| Mindsets can positively impact an individual’s: perception of our abilities & intelligence | 10 | 13 | 0.351 (0.135-0.568) | .68 | 0.38 | .073 |
| Mindsets can positively impact an individual’s: goal-oriented behaviours | 10 | 13 | -0.018 (-0.415-0.380) | .931 | -0.018 | .935 |
| Mindsets can positively impact an individual’s: motivation | 10 | 13 | 0.042 (-0.26-0.343) | .804 | 0.081 | .714 |
| Mindsets can positively impact an individual’s: visual scanning and perception | 10 | 13 | 0.20 (-0.07-0.469( | .328 | 0.274 | .206 |
| Mindsets can positively impact an individual’s: decision making | 10 | 13 | 0.017 (-0.318-0.352) | .930 | 0.01 | .964 |
| Mindsets can positively impact an individual’s: focus & attention | 10 | 13 | 0.307 (-0.041-0.654) | .100 | 0.357 | .094 |
| Mindsets can positively impact an individual’s: technical skill execution | 10 | 13 | 0.306 (-0.147-0.759) | .134 | 0.302 | .162 |
| Mindsets can positively impact an individual’s: physiological and psychological response to high-pressure situations | 10 | 13 | -0.15 (-0.326-0.026) | .426 | -0.182 | .407 |
| Are mindsets more fixed (cannot be changed) or more malleable (can be changed)? | 10 | 13 | 0.573 (0.257-0.890) | .005 | 0.534** | .009 |
| Is mindset important in the final few minutes BEFORE a high-pressure situation? ^1^ | 10 | 13 | There was 100% agreement between retest and original test. Weighted kappa is undefined due to no variation. | | | |
| DURING high-pressure situations is mindset important in optimising technical skill execution | 10 | 13 | 0.378 (0.147-0.609) | .028 | 0.502* | .015 |
| DURING high-pressure situations is mindset important in optimising tactical performance (e.g. decision making, problem solving) | 10 | 13 | 0.439 (0.028-0.85) | .024 | 0.362 | .089 |
| DURING high-pressure situations is mindset important in optimising physical performance | 10 | 13 | 0.767 (0.539-0.994) | .000 | 0.507* | .013 |
| In your professional career have you ever received mindset-specific education? ^1^ | 10 | 13 | 0.77 (0.553-0.987) | .000 | 0.771** | <.001 |
| κ_q:_ quadratic weighted kappa coefficient, ^1^ multiple-choice closed-ended question with three response categories (yes, no, unsure), PSS: Performance Support Staff. 95% CI: 95% confidence interval, spearman’s rho: spearman’s rank order correlation coefficient (2-tailed), * correlation is significant at the 0.05 level (2-tailed), ** correlation is significant at the 0.01 level (2-tailed) | | | | | | |

Categorical data was evaluated for level of agreement using a quadratic weighted Kappa (κ_q_) coefficient (Aldridge et al., 2017; Bilberg et al., 2014; de Raadt et al., 2021). Assessing reliability with κ_q_ mirrors the approach used in professional sport and military research (Kierkegaard et al., 2023; Querido et al., 2021) whilst simultaneously acknowledging that larger disagreements on Likert-scale response are disproportionally more meaningful than smaller changes (de Raadt et al., 2021). Classifications for reporting κ_q_ were <0.00 (poor), 0.00-0.20 (slight), 0.21-0.40 (fair), 0.41-0.60 (moderate), 0.61-0.80 (substantial) and 0.81-1.00 (almost perfect) (Landis & Koch, 1977). To measure the strength and direction of the relationships between non-parametric ordinal test-retest datasets a Spearman’s rank-order correlation (spearman’s rho) was utilised (de Raadt et al., 2021; Statistics, 2018; Vincent & Weir, 2012). The use of spearman’s rho is well established in published literature (Bargholtz et al., 2023; Bauhaus et al., 2023) Correlation coefficient range from +1 (perfect positive) to -1 (perfect negative) with a coefficient of zero indicating no association (Statistics, 2018). Classifications for reporting spearman’s rho were 0.00-0.19 (very weak), 0.20-0.39 (weak), 0.40-0.59 (moderate), 0.60-0.79 (strong) and 0.80-1.00 (very strong) (Bargholtz et al., 2023; Flora et al., 2023; Jess et al., 2024).

**References**

Aldridge, V. K., Dovey, T. M., & Wade, A. (2017). Assessing test-retest reliability of psychological measures: Persistent methodological problems. *European Psychologist*, *22*(4), 207.

Bargholtz, M., Brosved, M., Heimburg, K., Hellmark, M., Leosdottir, M., Hagströmer, M., & Bäck, M. (2023). Test–Retest Reliability, Agreement and Criterion Validity of Three Questionnaires for the Assessment of Physical Activity and Sedentary Time in Patients with Myocardial Infarction. *International journal of environmental research and public health*, *20*(16), 6579. <https://www.mdpi.com/1660-4601/20/16/6579>

Bauhaus, H., Jensen, P. M., Braun, H., & Thevis, M. (2023). Evaluation of Validity and Reliability of a German General and Sports Nutrition Knowledge Questionnaire for Athletes and Coaches (GSNKQ-AC). *Nutrients*, *15*(22). <https://doi.org/10.3390/nu15224844>

Bilberg, R., Nargaard, B., Roessler, K. K., & Overgaard, S. (2014). Test-retest reliability of Common Mental Disorders Questionnaire. *BMC Psychology*, *2*. <https://doi.org/10.1186/s40359-014-0032-5>

de Raadt, A., Warrens, M. J., Bosker, R. J., & Kiers, H. A. L. (2021). A Comparison of Reliability Coefficients for Ordinal Rating Scales. *Journal of Classification*, *38*(3), 519-543. <https://doi.org/10.1007/s00357-021-09386-5>

Flora, S., Marques, A., Hipólito, N., Morais, N., Silva, C. G., Januário, F., Rodrigues, F., Carreira, B. P., & Cruz, J. (2023). Test-retest reliability, agreement and construct validity of the International Physical Activity Questionnaire short-form (IPAQ-sf) in people with COPD. *Respiratory Medicine*, *206*, 107087. <https://doi.org/https://doi.org/10.1016/j.rmed.2022.107087>

Jess, L., Jarfelt, M., & Bäck, M. (2024). Reliability and validity of self-report questions for assessing levels of physical activity and sedentary time in adult childhood cancer survivors. *BMC Sports Science, Medicine and Rehabilitation*, *16*(1), 66. <https://doi.org/10.1186/s13102-024-00851-3>

Kierkegaard, M., Tegern, M., Broman, L., Halvarsson, A., & Larsson, H. (2023). Test-Retest Reliability and Translation of the Musculoskeletal Screening Protocol Questionnaire Used in the Swedish Armed Forces. *Mil Med*, *188*(7-8), 2318-2324. <https://doi.org/10.1093/milmed/usac082>

Landis, J. R., & Koch, G. G. (1977). The Measurement of Observer Agreement for Categorical Data. *Biometrics*, *33*(1), 159-174. <https://doi.org/10.2307/2529310>

Querido, S. M., Brito, J., Figueiredo, P., Carnide, F., Vaz, J. R., & Freitas, S. R. (2021). Post-match Recovery Practices in Professional Football: Design, Validity, and Reliability of a New Questionnaire. *Frontiers in sports and active living*, *3*, 680799. <https://doi.org/10.3389/fspor.2021.680799>

Statistics, L. (2018). *Spearman's correlation using SPSS Staistics. Statistical tutorials and software guides.* <https://statistics.laerd.com/>

Vincent, W. J., & Weir, J. P. (2012). *Statistics in Kinesiology* (4th Edition ed.). Human Kinetics.
